# MDI1228, a topical pan-JAK inhibitor, disrupts dermal fibroblast-T cell chemokine crosstalk to resolve allergic contact and atopic dermatitis

**DOI:** 10.3389/fimmu.2026.1875876

**Published:** 2026-07-20

**Authors:** Youxi Liu, Meimei Yin, Yichun Yang, Shujun Heng, Wenlu Zhang, Jinqiu Song, Binxing Feng, Yihan Yang, Wenjie Liu, Ling-juan Zhang

**Affiliations:** 1 State Key Laboratory of Cellular Stress Biology, School of Pharmaceutical Sciences, Xiamen University, Xiamen, China; 2 Dermatology Hospital of Jiangxi Province, Jiangxi Provincial Clinical Research Center for Skin Diseases, Candidate Branch of National Clinical Research Center for Skin Diseases, JXHC Key Laboratory of Skin Infection and Immunity, The Affiliated Dermatology Hospital of Nanchang University, Nanchang, Jiangxi, China; 3 Clinical Center for Biotherapy, Zhongshan Hospital (Xiamen), Fudan University, Xiamen, Fujian, China

**Keywords:** allergic contact dermatitis, atopic dermatitis, chemokine signaling axis, dermal fibroblast, JAK-STAT signaling pathway, MDI1228, T cell

## Abstract

**Introduction:**

Allergic contact dermatitis (ACD) and atopic dermatitis (AD) are driven by distinct T cell programs, and safe long-term topical therapies remain limited. Dermal fibroblasts (dFBs) have emerged as active immunomodulators, but whether they can be therapeutically targeted remains unexplored.

**Methods:**

Here we developed MDI1228, a novel topical pan‑JAK inhibitor with nanomolar potency against JAK1/2/3/TYK2 (IC₅₀ 0.11-0.85 nM) and high selectivity. MDI1228 was evaluated in DNFB‑induced ACD and MC903‑induced AD mouse models, as well as in primary mouse and human cell‑based assays.

**Results:**

Topical MDI1228 ameliorated both ACD and AD in mice, reducing T cell infiltration and cytokine production. Mechanistically, MDI1228 not only directly inhibited T cell activation and cytokine production but also disrupted fibroblast‑T cell crosstalk by reducing dFB‑derived chemokine expression. Single‑cell transcriptomics identified dFBs as the primary source of CXCL9/10 in ACD and CCL2 in AD. Conditioned medium and neutralization experiments demonstrated that CXCL9/10‑CXCR3 and CCL2‑CCR2 signaling axes contribute to T cell polarization in a context‑dependent manner. Compared with glucocorticoids, prolonged topical application of MDI1228 showed minimal systemic toxicity and preserved tissue homeostasis.

**Discussion:**

These findings identify dFBs as a central therapeutic node and demonstrate that MDI1228, by directly targeting T cells and disrupting dFB‑derived chemokine axes via JAK inhibition, offers a potent and safe topical treatment for both ACD and AD.

## 1 Introduction

Allergic contact dermatitis (ACD) and atopic dermatitis (AD) are common, chronic inflammatory skin diseases that impose a major burden on quality of life and healthcare resources. ACD is a delayed-type hypersensitivity reaction driven by hapten-specific CD8+ cytotoxic T (Tc1) cells producing IFNγ (1, 2), whereas AD is dominated by Th2-type immunity with IL4/IL13-mediated barrier dysfunction and pruritus (3, 4). Despite divergent immune signatures, both diseases progress through reciprocal fibroblast-immune cell circuits that amplify and perpetuate cutaneous inflammation (5, 6).

Although pro-inflammatory factors have traditionally been attributed to keratinocytes and infiltrating immune cells in dermatitis, emerging evidence suggests that non-hematopoietic stromal cells may also play a critical role in shaping the inflammatory microenvironment. Dermal fibroblasts (dFBs) are increasingly recognized as active immunomodulators rather than passive structural elements. Activated by IFNγ or IL4/IL13, dFBs secrete chemokines (e.g. CXCL9/10, CXCL1/12, CCL2), cytokines (e.g. IL6, IL8), and extracellular-matrix remodelling factors that recruit and retain pathogenic T cells, neutrophils and macrophages, creating self-sustaining inflammatory loops (5–12). Targeting dFB signaling therefore represents an unexplored therapeutic opportunity.

Topical glucocorticoids (GCs) remain first-line therapy for ACD/AD. They rapidly suppress inflammation by trans-repressing NF-κB and AP-1, thereby silencing multiple cytokines (e.g. IL1β, TNFα, IL2, IL4, IFNγ) (13). Because NF-κB sits at the apex of both innate and adaptive inflammatory cascades, GCs provide broad, non-specific immunosuppression that quickly reduces erythema, oedema and pruritus. However, this “upstream” mode of action is also their major weakness: prolonged or high-potency topical GC use causes well-documented local adverse events including epidermal atrophy, telangiectasia, striae, delayed wound healing, steroid acne and rosacea-like eruptions (14).

The Janus kinase (JAK)-STAT pathway represents a more targeted therapeutic node. Cytokines central to ACD (e.g. IFNγ, IL2, IL15) and AD (e.g. IL4, IL13, IL31) signal exclusively through JAK1/JAK2/JAK3/TYK2; thus pan-JAK inhibition can interrupt disease-specific circuits without global NF-κB blockade (15, 16). Proof-of-concept has been established with topical tofacitinib and delgocitinib in AD, and with oral baricitinib/upadacitinib for moderate-to-severe AD (17, 18). However, available topical agents are either JAK1-selective or have not been evaluated in ACD, and none have been optimized to suppress cytokine-activated dermal fibroblasts that sustain chronic inflammation.

In this study, we developed MDI1228, a novel small-molecule pan-JAK inhibitor with nanomolar potency against JAK1/2/3 and TYK2 and a favorable safety profile. MDI1228 was formulated as a stable, cosmetically acceptable hydrogel. Using DNFB-induced ACD and MC903-induced AD mouse models, we identified dFBs as a predominant source of key chemokines, specifically CXCL9/10 in ACD and CCL2 in AD. MDI1228 not only directly inhibits T cell activation, but also suppressed these dFB-derived chemokine axes, thereby disrupting fibroblast-T cell crosstalk and limiting T cell polarization in lesional skin. These findings reveal an underappreciated role of dFBs as central regulators of pathogenic T cell differentiation and highlight the therapeutic potential of targeting dFB-derived chemokine axes via JAK inhibition in both ACD and AD.

## 2 Materials and methods

### 2.1 Animals and animal cares

The C57BL/6 mice used in this study were purchased from GemPharmatech (Nanjing, China) and bred under specific pathogen-free (SPF) conditions at the Xiamen University Laboratory Animal Center. All animal experiments were conducted in accordance with the relevant standards of the Xiamen University Animal Care and Use Committee (Protocol No. XMULAC20240069).

### 2.2 Preparation and toxicokinetic studies of MDI1228 and blank hydrogel

MDI1228 hydrogel (0.5% and 1%) was prepared by dissolving disodium edetate, anhydrous sodium sulfite, glycerin, and vitamin C in purified water to form “Solution 1”, with 10% reserved for container rinsing. Chlorobutanol was dissolved in 5.0% of the propylene glycol to obtain “Solution 2”. The remaining propylene glycol and Solution 2 were transferred to a homogenizing emulsifier, containers were rinsed with reserved Solution 1, and the rinsate was added. Sieved MDI1228 was incorporated under homogenization until uniform. Solution 1 was added under homogenization, followed by a portion of triethanolamine, and the mixture was homogenized under vacuum. Carbomer ETD2020 NF was added portionwise, homogenized under vacuum, and stirred continuously under vacuum for at least 13 h to ensure complete polymer swelling. The remaining triethanolamine, diluted with the final portion of propylene glycol, was added to adjust pH to 5.8~6.8. The final gel was defoamed under vacuum with stirring. Blank hydrogel was prepared identically without MDI1228.

Toxicokinetic studies were conducted in 7~9-week-old Sprague-Dawley rats receiving once-daily topical doses of 20, 40, or 120 mg/kg MDI1228 for 14 days. Blood and skin samples were collected at designated time points for LC-MS/MS analysis of MDI1228 concentrations. In parallel, safety assessments were performed including body weight, hematology (white blood cell count, WBC), serum biochemistry (alanine aminotransferase, ALT; aspartate aminotransferase, AST), Draize score for local skin irritation, gross necropsy, and absolute and relative organ weights (heart, liver, spleen, kidneys). Draize scoring was performed at each application site using a 0–4 scale for erythema/edema (0 = none, 1 = very slight, 2 = well defined, 3 = moderate to severe, 4 = severe). All evaluations were conducted by Pharmaron Inc. (TSP project code: 03303-201175). Dose conversions between species were performed using the FDA-recommended body surface area normalization method, applying the standard Km factors (mouse: 3, rat: 6, human: 37) (19).

### 2.3 Kinase inhibition profiling of MDI-1228

MDI1228 was dissolved in DMSO to 100× the final concentration and diluted 25−fold with assay buffer (20 mM HEPES, 0.01% Triton X−100, 1 mM DTT, pH 7.5) to a working concentration of 0.2 μM. Kinase reactions were performed in 384−well plates by mixing 5 μL of 4× compound, 5 μL of 4× substrate/ATP/metal solution (20 mM HEPES, 0.01% Triton X−100, 5 mM DTT, pH 7.5), and 10 μL of 2× kinase solution. After incubation at room temperature for 1 or 5 hrs. (kinase−dependent), the reaction was terminated with 70 μL of termination buffer (Carna Biosciences). Product and substrate peptides were separated and quantified using a LabChip™ system (Perkin Elmer). Percent inhibition was calculated with the complete reaction mixture as 0% inhibition and the enzyme−free control as 100% inhibition.

### 2.4 *In vitro* JAK kinase inhibition assay of MDI1228

MDI1228 inhibitory activity against JAK1, JAK2, JAK3, and TYK2 was determined using the ADP-Glo™ kinase assay (Promega, #V9101). Test compounds (MDI1228 and filgotinib) were serially diluted in 100% DMSO in 384-well dilution plates (Labcyte, #PP-0200) and transferred (0.1 μL/well) into assay plates (PerkinElmer, #6007290) using an Echo acoustic dispenser (Labcyte, #550). Kinase solutions prepared in reaction buffer (50 mM HEPES, 10 mM MgCl_2_, 0.01% Brij-35, 1 mM EGTA, 2 mM DTT) were added (5 μL/well) and pre-incubated for 15 mins at 25 °C. Reactions were initiated by adding 5 μL of substrate mixtures containing ATP and specific peptides (IRS1 for JAK1/TYK2, IGF1Ride for JAK2, or Poly (4:1 Glu, Tyr) for JAK3). After 60 mins incubation at 25 °C, 10 μL ADP-Glo™ reagent was added for 40 mins, followed by 20 μL detection reagent for another 40 mins. Luminescence (RLU) was measured using an Envision plate reader (PerkinElmer, #2104). IC_50_ values were calculated by four-parameter logistic curve fit. Z’ factors > 0.7 confirmed assay quality for all kinases.

### 2.5 Establishment of DNFB-induced ACD and MC903-induced AD mouse model

The DNFB-induced ACD mouse model was established following a previously described protocol (20). For sensitization, 25 μL of 1.0% DNFB (dissolved in acetone and olive oil at a 4:1 ratio; Rhawn, #R010240) was applied to the abdominal skin of 8-week-old male mice (n=10/group). Six days later, ACD was elicited by applying 20 μL of 0.2% DNFB to the dorsal skin. Dorsal skin tissues were collected at 60 hrs. after elicitation for further analysis. For therapeutic intervention, 50 mg 0.5% or 1% MDI1228 was topically applied to the dorsal skin twice daily (25 or 50 mg/kg/day) from 12 hrs. post-elicitation. A 1% hydrocortisone (HC) ointment (Aveeno, UK) was used as a positive control, while blank hydrogel served as the vehicle control. The Eczema Area and Severity Index (EASI) score for mouse dorsal skin was assessed by a blinded observer based on the sum of scores for erythema, edema/papulation, excoriation, and lichenification, each graded on a scale of 0 (none) to 3 (severe).

Atopic dermatitis (AD)-like skin inflammation was triggered by topical application of 45μL MC903 (100μM, MCE, #HY-10001) once a day from day 0 to day 10 on dorsal skin, and dorsal skin samples were collected at day 10 for analysis. For treatments, 50 mg 0.5% or 1% MDI1228 was topically applied to dorsal skin once a day (12.5 or 25 mg/kg/day) from day4 to day 9. 1% HC ointment was used as the positive drug, and blank hydrogel was used as the vehicle control. The SCORAD index (SCORing atopic dermatitis) was assessed by a blinded observer based on the sum of scores for erythema, edema/papulation, excoriation, and lichenification, each graded on a scale of 0 (none) to 3 (severe).

### 2.6 Bulk RNA sequencing and bioinformatic analysis

For RNA extraction and quality assessment, total RNA was isolated using TRIzol reagent (Sigma, #T9424) in combination with the RNAExpress Total RNA Kit (NCM, #M050). RNA integrity was evaluated using a bioanalyzer, and only samples with an RNA Integrity Number (RIN) greater than 7 were selected for sequencing.

Sequencing libraries were constructed from poly(A)-enriched RNA using the NEBNext Ultra RNA Library Preparation Kit (for Illumina) following the manufacturer’s protocol. The procedure encompassed mRNA fragmentation, cDNA synthesis, end repair and A-tailing, adapter ligation, and size selection targeting insert fragments of approximately 300 bp. After quality and quantity verification, the libraries were subjected to 2×150 bp paired-end sequencing on the Illumina NovaSeq platform.

Bioinformatics analysis was conducted by GENEWIZ for raw data processing. Venn diagrams were generated using BioVenn software, and KEGG enrichment analysis of differentially expressed genes was performed using OmicShare Tools (21).

### 2.7 Single-cell RNA sequencing and data analysis

Single-cell suspensions were prepared from mouse dorsal skin samples as previously described (6). Briefly, skin was minced and digested with 0.25% collagenase IV (Gibco) and 0.1% DNase I (Sigma) for 1 hr. at 37 °C. The resulting cell suspension was filtered through a 40 μm strainer, and red blood cells were removed using ACK lysis buffer. Viability (>85%) was confirmed by trypan blue exclusion. Libraries were constructed using the Chromium Next GEM Single Cell 3' Reagent Kit v3.1 (10x Genomics) according to the manufacturer’s protocol. Sequencing was performed on an Illumina NovaSeq 6000 platform with a target depth of 50,000 reads per cell. Raw sequencing data were processed with Cell Ranger (v6.0.0) using the mouse reference genome (mm10-2020-A). Downstream analysis including quality control, dimensionality reduction, clustering, and marker gene identification was performed using the Seurat package (v4.3.0) in R. Cells with <200 or >6000 detected genes, or >10% mitochondrial reads, were excluded. Clusters were annotated based on canonical marker genes (e.g., *Pdgfra* for fibroblasts, *Cd3e* for T cells, *Cd68* for macrophages). Cell-cell communication was inferred using CellChat (v1.6.0) with default parameters.

### 2.8 Flow cytometry analysis

The procedure for flow cytometric analysis of T cells was adapted from previously established protocols (22). To evaluate cytokine production in inhibitor-treated T lymphocytes, isolated skin-draining lymph node T cells were treated as described and incubated with PMA (50 ng/mL, Yeasen, China) and ionomycin (500 ng/mL, Sigma-Aldrich, USA) for 2 hours, followed by the addition of a Golgi transport inhibitor (1:1000, BD Biosciences, USA) for another 1 hr. before staining. Viability was assessed using Zombie Violet™ Fixable Viability Kit (BioLegend, #423114) to exclude dead cells. After Fc receptor blockade with anti-mouse CD16/32 antibody (eBioscience, #14016185), surface staining was performed using PECy7-conjugated anti-CD45 (BioLegend, #147704), PerCP-Cy5.5-conjugated anti-CD8 (eBioscience, #45-0081-82), and APC-conjugated anti-CD4 (BioLegend, #100516). For intracellular cytokine detection, cells were fixed and permeabilized using the Intracellular Fixation and Permeabilization Buffer Kit (eBioscience, #00-8333-56) and subsequently stained with FITC-conjugated anti-IL4 (BioLegend, #504109), FITC-conjugated anti-IL13 (eBioscience, #53-7133-82), AF700-conjugated anti-IFNγ (BioLegend, #505824), and PE-conjugated anti-IL17A (eBioscience, #12-7177-81).

Data acquisition was carried out on a Thermo Attune NxT flow cytometer, and all analyses were performed using FlowJo V10 software. Cells positive for Zombie Violet dye were excluded from the final analysis to ensure data accuracy.

### 2.9 Histology, Masson, EVG-Weigert, and immunohistochemistry staining

Immunohistochemical staining was performed on frozen sections as previously described (22). Hematoxylin-eosin (HE) staining was used for histological assessment. Collagen and elastic fibers were visualized using Masson’s trichrome and EVG-Weigert staining kits (Solarbio, China), respectively. For immunostaining, sections were fixed, permeabilized with 0.1% saponin (Sigma-Aldrich), and blocked with 5% BSA. Primary antibodies were incubated overnight at 4 °C, followed by fluorophore-conjugated secondary antibodies (AF488, Cy5, or Cy3; 1:250; Jackson Immunological Research) for 4 h at 4 °C in the dark. Sections were mounted with ProLong Gold antifade reagent containing DAPI (Thermo Fisher). Images were acquired using an Aperio VERSA scanner or Leica TCS SP8 confocal microscope and analyzed with Photoshop and Aperio ImageScope software.

Primary antibodies used were: PE-conjugated anti-CD8a (1:100, BioLegend, #100708); rabbit anti-CD4 (1:100, CST, #25229S); Alexa Fluor^®^ 488-conjugated anti-IFNγ (1:100, BioLegend, #505813); Alexa Fluor^®^ 488-conjugated anti-IL4 (1:100, BioLegend, #504109); rabbit anti-pSTAT1 (1:100, CST, #9167S); and rabbit anti-pSTAT6 (1:100, CST, #56554S). Secondary antibodies (Jackson Immunological Research) included Cy3- or AF647-conjugated anti-rabbit IgG, Cy3- or AF647-conjugated anti-goat IgG, and AF647-conjugated anti-rat IgG, all used at 1:250.

For quantitative histopathological analysis of HE-stained sections, three non-overlapping fields were randomly selected from each section. Epidermal acanthosis was measured as the vertical distance from the basal membrane to the outermost layer of the stratum granulosum using ImageJ software. Dermal thickness was defined as the distance from the basement membrane to the upper border of the subcutaneous adipose tissue. dWAT thickness was measured as the vertical height of the subcutaneous white adipose tissue layer. Cellular infiltration density in the dermis and dWAT was quantified by counting the number of infiltrating inflammatory cells per square millimeter using ImageJ. Dermal edema was evaluated using a semiquantitative scoring system on a 0 to 4 scale based on interstitial fluid accumulation and collagen fiber separation. All quantifications were performed in a blinded manner, with the observer unaware of the treatment group assignments.

### 2.10 Quantitative reverse transcription-quantitative PCR analyses

RNA isolation from total cellular content was performed with the RNA Express Total RNA Kit (NCM, Suzhou, China). For cDNA synthesis, 50 ng of RNA was reverse-transcribed using the HiScript III Q RT SuperMix Kit (Vazyme, China). Quantitative real-time PCR was subsequently carried out on a Qtower real-time system (Analytik Jena, Germany) employing SYBR Green Mix (Bimake, Houston, Texas, USA). To avoid non-specific amplification from genomic DNA, all SYBR Green primers were deliberately designed to span at least one exon-exon junction. Gene expression levels were normalized to the housekeeping genes *Tbp* or *Rplp0*. The primer sequences employed are provided in **Supplementary Table 1**.

### 2.11 Cell extract preparation and ELISA assay

To ensure protein integrity and prevent degradation, skin biopsy samples or cells were lysed in buffer supplemented with a complete protease inhibitor cocktail (Apexbio, USA), following a previously described protocol (23). The lysates were homogenized using a Digital Sonifier FS-350T ultrasonic processor (Sxsonic, China) and subsequently centrifuged to remove DNA and cellular debris. Protein concentrations were determined using a BCA Protein Assay Kit (Thermo, #A65453). For enzyme-linked immunosorbent assay (ELISA), protein lysates were diluted into 50μg per 100 μL and processed with the Mouse DuoSet^®^ ELISA Kit (R&D Systems, USA) according to the manufacturer’s instructions.

### 2.12 Cell extract preparation and western blotting analyses

Western blot analysis was performed on skin biopsy specimens or cultured cells lysed in denaturing buffer (20 mM HEPES pH 7.4, 250 mM NaCl, 2 mM EDTA, 1% SDS) supplemented with protease and phosphatase inhibitor cocktails (Apexbio, Roche) as described (24). Lysates were boiled for 3 mins, sonicated (FS-350T, Sxsonic), and centrifuged to remove debris. Protein concentrations were determined by BCA assay (Thermo Fisher). For Western blot, 20 μg protein was mixed with 2× loading buffer (Sigma-Aldrich) containing 20% β-mercaptoethanol, denatured at 95 °C for 5 mins, resolved on 10–20% Tris-Tricine precast gels (Thermo Fisher), and transferred to PVDF membranes (Roche). Membranes were probed with primary antibodies overnight at 4 °C, followed by fluorescent secondary antibodies (LI-COR). Bands were visualized using the Odyssey Imaging System (LI-COR). All blots are representative of three independent experiments.

Primary antibodies used: rabbit anti-total STAT1 (CST, #9172S), rabbit anti-total STAT6 (CST, #9362S), mouse anti-β-actin (Proteintech, #66009-1-Ig). Secondary antibodies: IRDye^®^ 680RD donkey anti-mouse IgG (LI-COR, #926-68072) and IRDye^®^ 800CW donkey anti-rabbit IgG (LI-COR, #926-32213). All antibodies were used at manufacturer-recommended dilutions. Additional primary antibody details are provided in Method 2.9.

### 2.13 Primary culture of mouse or human dermal fibroblasts

Primary mouse dFBs were isolated from murine skin as described (22). Neonatal human dFBs (back skin) were obtained from IIAM (Exton, PA). Fibroblasts were cultured in DMEM with 10% FBS and antibiotics at 37 °C with 5% CO_2_. For experiments, first-passage cells were plated at 1×10^5^ cells/mL, pre-incubated with 0.01-10 μM MDI1228, dexamethasone (Selleck, #S1322), tofacitinib (bidepharm, #BD33742), or delgocitinib (bidepharm, #BD00778677) for 30 mins (DMSO as control), and then stimulated for 24 hrs. with recombinant cytokines: mouse IFNγ (5 ng/mL, #485-MI-100), mouse IL4 (5 ng/mL, #404-ML-010), human IFNγ (5 ng/mL, #285-IF-100), or human IL4 (10 ng/mL, #204-IL-050). All cytokines were from R&D Systems. Supernatants, cell lysates, and RNA were collected for ELISA and qRT-PCR analysis.

### 2.14 Primary T cell culture and dermal fibroblast conditioned medium-T cell co-culture

For T cell culture, primary lymphocytes from skin-draining lymph nodes of adult C57BL/6 mice were seeded onto plates with or without pre-coated anti-CD3 (3 μg/ml, Biolegend, #100340) and anti-CD28 (3 μg/ml, Biolegend, #102116). Cells were cultured in RPMI with 10% FBS and 1% antibiotics. For treatment, T cells were exposed to 1 μM MDI1228, dexamethasone, or tofacitinib (DMSO as control). After 5 days, supernatants and cells were collected for ELISA and flow cytometry.

For collection of fibroblast-conditioned medium (CM), primary dFBs were pre-incubated with 5 μM MDI1228, dexamethasone, tofacitinib, or delgocitinib (DMSO as control) for 30 mins, then stimulated with IFNγ (10 ng/ml), IL4 (20 ng/ml), or PBS for 2 hrs., washed twice with PBS, replenished with fresh medium, and incubated for 48 hrs. before CM collection. As a drug-only washing control, MDI1228 was added to cell-free wells and subjected to the same washing and medium change steps, after which the resulting medium was used for T cell culture to exclude the possibility that trace amounts of residual MDI1228 after washing could enter the conditioned medium.

For co-culture, T cells were first pre-incubated with 20 μg/mL of neutralizing antibody against CXCR3 (BioLegend, #126513), or against CCL2 (BioLegend, #505902) for 30 mins, with the corresponding IgG isotype control (BioLegend, #400940). After incubation, cells were then cultured in 1:1 dFB-CM: RPMI medium. After 5 days, supernatants and cells were collected for ELISA and flow cytometry.

### 2.15 Human T cell isolation and culture

Human T cells isolated from peripheral blood mononuclear cells (Milecell Biotechnologies, #PB100C) were cultured in RPMI with 10% FBS and 1% antibiotics. Activation was achieved using CD3/CD28 Dynabeads (1:10, Thermo Fisher, #11161D) plus human IL2 (10 ng/mL, Novoprotein, #C013) and IL7 (10 ng/mL, Novoprotein, #CX47) for 5 days. For treatment, activated T cells were exposed to 1 μM MDI1228, dexamethasone, or tofacitinib (DMSO as control). After 5 days, supernatants and cells were collected for ELISA and qRT-PCR.

For conditioned medium collection, hdFBs were pre-stimulated with hIFNγ (20 ng/mL), hIL4 (5 ng/mL), or PBS for 2 hrs., washed twice with PBS, replenished with fresh medium, and incubated for 48 hrs. before CM collection. For co-culture, human T cells were pre-incubated with 1 μM MDI1228, dexamethasone, or tofacitinib for 30 mins, then cultured in 1:1 hdFB-CM: RPMI medium. After 5 days, supernatants and cells were collected for ELISA and qRT-PCR.

### 2.16 Statistical analysis

All experiments in this study were performed a minimum of three independent times, and statistical analyses were conducted using GraphPad Prism 10 software. For comparisons between two groups, statistical significance was assessed using an unpaired two-tailed Student’s t-test. For experiments involving multiple groups, one-way or two-way analysis of variance (ANOVA) was employed for multiple comparisons. A P value of less than 0.05 was considered statistically significant (*P < 0.05, **P < 0.01, ***P < 0.001, ****P < 0.0001).

## 3 Result

### 3.1 Physicochemical properties and pharmacological activity evaluation of MDI1228

**Figure 1:**
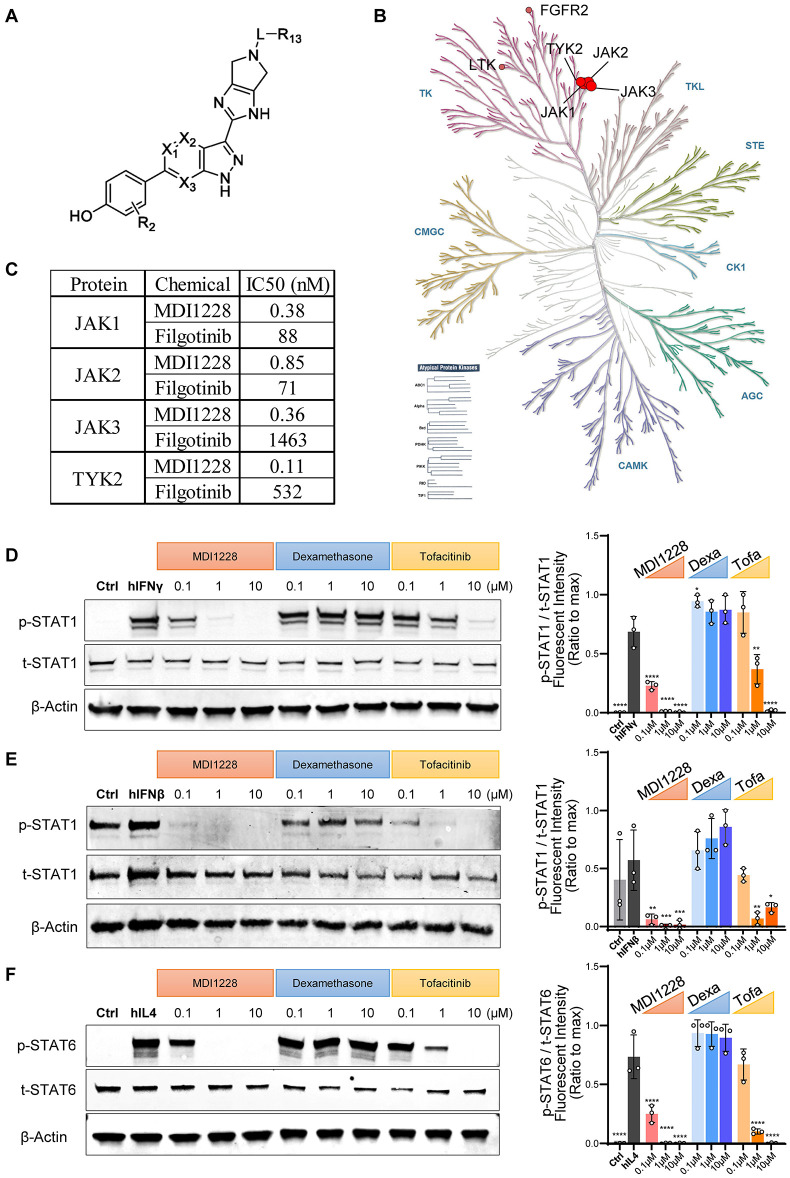
Physicochemical properties and pharmacological activity evaluation of MDI1228. **(A)** Chemical structural formula of MDI1228. **(B)** Kinase selectivity profile of MDI1228. Measurement of 148 kinases was performed at 0.2 μM of MDI1228 and only kinases with an inhibition rate > 90% are shown. The inhibition rate is encoded by the size of red circles. This plot was generated by the online tool KinMap (http://kinhub.org/kinmap/). **(C)** Inhibitory activity evaluation of MDI1228 on JAK protein. 0–1000 nM of MDI1228 or Filgotinib were mixed with JAK1, JAK2, JAK3, and TYK2 substrates, and the RLU signal intensity was measured and the IC_50_ for JAK inhibition activity by JAK inhibitors was calculated. **(D–F)** Human dFBs were pre-treated with MDI1228, tofacitinib or dexamethasone (0.1 μM, 1 μM, or 10 μM) for 30 min and then treated with hIFNγ (in D), hIFNβ (in E) or hIL4 (in F) for 1 hr. Control or cytokine-treated samples were subjected to western blot analysis of STAT1 or STAT6 phosphorylation levels (n=3/group). Quantified results were shown on the right panel. All error bars indicate mean ± SEM. *p < 0.05, **p < 0.01, ***p < 0.001, ****p < 0.0001.

To develop an efficacious and safe topical treatment for ACD and AD, we designed a novel pan-JAK inhibitor, MDI1228, formulated as a hydrogel (**Figure 1A**). To assess kinase selectivity, we profiled MDI1228 against a panel of 148 kinases at 0.2 µM. Among these, MDI1228 inhibited only four kinases (JAK1, JAK2, JAK3, TYK2) by more than 95%, and two additional kinases (FGFR2 and LTK) by more than 90% (**Figure 1B**; **Supplementary Table 2**). Nevertheless, GSEA analysis of differentially expressed genes in mouse skin treated with MDI1228 versus blank hydrogel for two weeks revealed no significant enrichment of FGFR or cell surface receptor tyrosine kinase (RTK) signaling pathways (**Supplementary Figure 1A**). We therefore excluded the possibility that MDI1228 alleviates ACD and AD inflammation through targeting FGFR2 or LTK rather than the JAK-STAT signaling pathway. Pharmacological activity evaluation showed that MDI1228 potently inhibited JAK1, JAK2, JAK3, and TYK2 at extremely low concentrations, with IC50 values of 0.38, 0.85, 0.36, and 0.11 nM, respectively. This inhibitory potency was markedly superior to that of the classic JAK1 inhibitor filgotinib, which had IC50 values of 88, 71, 1463, and 532 nM for JAK1, JAK2, JAK3, and TYK2, respectively (**Figure 1C**).

Based on CCK-8 assays, 10 μM MDI1228 and tofacitinib showed toxicity to macrophages (Raw264.7) and T cells (EL4) but not to primary mouse dFBs. Consequently, we selected 10 μM as the highest concentration for dFB treatments and 1 μM for T cell treatments in subsequent experiments (**Supplementary Figure 1B**). Toxicokinetic evaluation in rats confirmed a safety profile of topical MDI1228. Following 14-day repeated dermal application, the maximum tolerated dose (MTD) was determined as 120 mg/kg/day. Systemic plasma exposure (C_max_ and AUC_last_) generally increased in a less-than-dose-proportional manner over the 20–120 mg/kg/day range. Noticeable drug accumulation occurred only at the highest dose (120 mg/kg/day in rats corresponds to a human equivalent topical dose of ~19.4 mg/kg/day) (19), which is far exceeds the projected human exposure (~1.84 mg/kg/day for topical 1.5% ruxolitinib twice a day) (25). At the MTD of 120 mg/kg/day, local skin concentrations reached extremely high levels (e.g., male: 51,367 ng/g; **Table 1**). Even with elevated systemic exposure in males at this dose (C_max_ 38.7 ng/mL, AUC_last_ 177 hr·ng/mL; accumulation ratio of 10.2) (**Table 2**), no drug-related changes were detected in hematological or biochemical parameters (including WBC, ALT, and AST), organ weights, or local skin reactions at any dose (**Supplementary Tables 3**–**5**). Collectively, these data suggest that topical MDI1228 is well tolerated with no detectable systemic or local toxicity under the tested conditions.

**Table 1:** Concentration of MDI1228 in rat skin homogenate following topical application.

Post-treatment day	Dose (mg/kg/day)	Sexual	Concentration of MDI1228 (ng/mL)	Concentration of MDI1228 in skin (ng/g)
Day15	20	Male	3820	19100
		Female	3540	17700
	40	Male	6013	30067
		Female	8583	42917
	120	Male	10273	51367
		Female	17000	85000

**Table 2:** Mean toxicokinetic parameters of MDI1228 in rat plasma following topical application.

Post-treatment day	Dose (mg/kg/day)	Sexual	C_max_ (ng/mL)	AUC_last_ (hr*ng/mL)
Day 1	20	Male	1.3	6.09
		Female	1.74	13.3
	40	Male	1.93	10.6
		Female	2.66	16.2
	120	Male	3.4	17.3
		Female	3.51	21.8
Day 14	20	Male	1.04	5.23
		Female	1.42	8.93
	40	Male	1.27	6.65
		Female	3.2	30.4
	120	Male	38.7	177
		Female	5.82	50.5
Day 14/Day 1	20	Male	0.796	0.86
		Female	0.815	0.674
	40	Male	0.661	0.627
		Female	1.2	1.88
	120	Male	11.4	10.2
		Female	1.66	2.31

C_Max_, maximum concentration; AUC_last_, area under the concentration-time curve (from the start to the last dosing time point).

Given the crucial role of dFBs in autoimmune dermatitis pathogenesis mediated by type I or type II cytokines (5, 6), we evaluated the anti-inflammatory effects of MDI1228 on dFBs stimulated by type I (IFNγ, IFNβ) and type II (IL4) cytokines. Western blot analysis showed that MDI1228 significantly suppressed STAT1 or STAT6 phosphorylation in human dFBs (hdFBs) stimulated by IFNγ, IFNβ, or IL4 at 0.1 µM. This inhibition reached 99% at 1 µM, outperforming the JAK1/3 inhibitor tofacitinib. In contrast, dexamethasone had no inhibitory effect on STAT phosphorylation (**Figures 1D–F**).

These results demonstrate that MDI1228 exhibits potent inhibitory activity against JAKs and suppresses type I/II cytokine-driven JAK-STAT signaling in human dFBs, without detectable cytotoxicity at the tested concentrations.

### 3.2 Topical MDI1228 ameliorates ACD- and AD-like skin inflammation in mice

**Figure 2:**
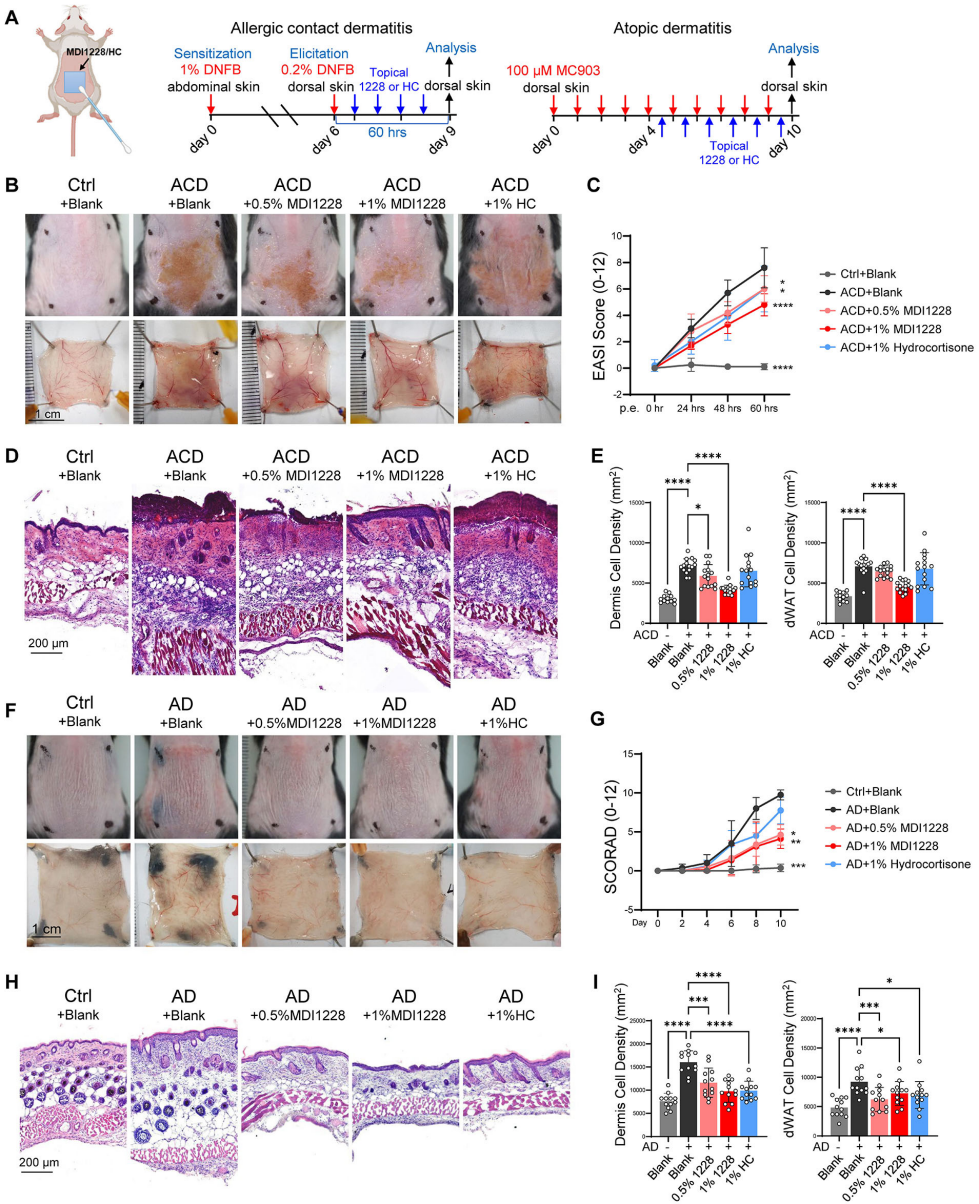
Topical MDI1228 ameliorates ACD- and AD-like skin inflammation in mice. **(A)** Overview of the experimental setting. ACD-like skin inflammation was triggered by sequential sensitization at Day 0 and elicitation at Day 6 of ACD by topical application of 1% or 0.2% DNFB on belly or dorsal skin as indicated. MDI1228 (0.5% or 1%), Hydrocortisone (1%) or blank hydrogel was topically applied on dorsal skin twice a day from 12 hrs to 48 hrs post-elicitation. Dorsal skin was collected at 60 hrs post-elicitation for analyses. AD-like skin inflammation was triggered by topical application of 100uM MC903 once a day from Day 0 to Day 10 on dorsal skin. MDI1228 (0.5% or 1%), Hydrocortisone (1%) or blank hydrogel was topically applied on dorsal skin once a day from Day 4 to Day 9. Dorsal skin was collected at Day 10 for analyses. **(B-E)** Representative dorsal skin images **(B)**, EASI scores **(C)**, H&E staining **(D)**, and the cell density of dermis or dWAT area **(E)** for each group at 60 hrs after ACD elicitation (n=4~5 or 12~15/group). **(F–I)** Representative dorsal skin images **(F)**, SCORAD index **(G)**, H&E staining **(H)**, and the cell density of dermis or dWAT area **(I)** for each group at Day 10 (n=4 or 12/group). All error bars indicate mean ± SEM. *p < 0.05, **p < 0.01, ***p < 0.001, ****p < 0.0001.

To evaluate the therapeutic efficacy of MDI1228, 0.5% or 1% MDI1228 hydrogel (~1.05 or 2.10 mM; 12.5–50 mg/kg/day in mice corresponding to ~6.25–25 mg/kg/day safe in rats) (19), 1% hydrocortisone ointment (~2.76 mM), or a blank hydrogel were applied topically in DNFB-elicited ACD-like and MC903-induced AD-like dermatitis mouse models (**Figure 2A**). In the ACD model, MDI1228 treatment significantly ameliorated DNFB-induced clinical phenotypes, including erythema, crusting, and angiogenesis, as assessed by skin phenotyping and EASI scores (**Figures 2B, C**). H&E staining revealed that MDI1228 markedly reduced inflammatory cell infiltration in the dermis and dWAT (**Figures 2D, E**) and significantly suppressed epidermal acanthosis in ACD mice, without significantly affecting dermal thickening or edema compared to the control group (**Supplementary Figures 2A, B**). In contrast, hydrocortisone treatment appeared to exacerbate the inflammatory response. Mouse body weight, spleen weight, and lymph node weight remained unaltered across all treatment groups in ACD (**Supplementary Figures 2C–E**).

In the AD model, both MDI1228 and hydrocortisone effectively improved MC903-induced erythema, wrinkling, and angiogenesis (**Figures 2F, G**). Histologically, MDI1228 and hydrocortisone showed comparable efficacy in reducing inflammatory infiltration in the dermis and dWAT, as well as in decreasing epidermal, dermal and dWAT thickness (**Figures 2H, I**; **Supplementary Figures 2F, G**). Notably, hydrocortisone- but not MDI1228-treated mice exhibited significantly reduced body weight and spleen weight compared to the control (**Supplementary Figures 2H–J**), indicating systemic immunosuppressive effects of hydrocortisone under the same treatment regimen.

In summary, MDI1228 significantly alleviated ACD-like inflammation, outperforming hydrocortisone, while both agents showed comparable efficacy in ameliorating AD-like dermatitis.

### 3.3 MDI1228 directly suppresses pathogenic T cell responses *in vitro* and *in vivo*

**Figure 3:**
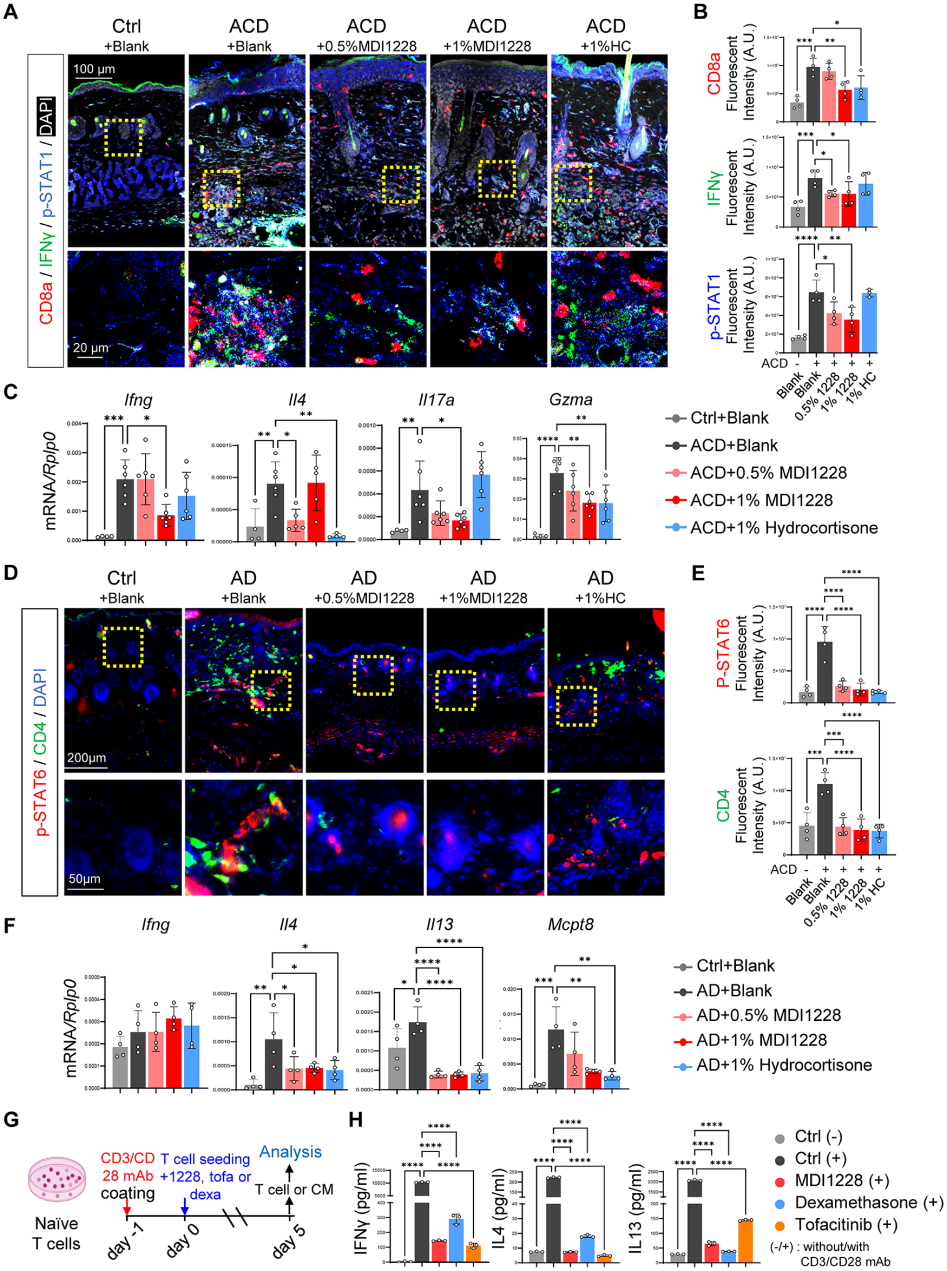
MDI1228 directly suppresses pathogenic T cell responses *in vitro* and *in vivo.* **(A, B)** Immunostaining **(A)** and the quantified fluorescent intensity **(B)** of ACD skin sections with anti-CD8a (red), anti-IFNγ (green) and anti-pSTAT1 (blue) antibodies, and nuclei were counter stained by DAPI (white). Zoom-in images were shown in the lower panel (n=4/group). **(C)** qRT-PCR analysis of the mRNA expression levels of indicated genes (ratios to HK gene *Rplp0* were shown, n=4~6/group). **(D, E)** Immunostaining **(D)** and the quantified fluorescent intensity **(E)** of AD skin sections with anti-pSTAT6 (red) and anti-CD4 (green) antibodies, and nuclei were counter stained by DAPI (blue). Zoom-in images were shown in the lower panel (n=4/group). **(F)** qRT-PCR analysis of the mRNA expression levels of indicated genes (ratios to HK gene *Rplp0* were shown, n=4/group). **(G)** Overview of the experimental setting. Naïve T lymphocytes stimulated with CD3/CD28 mAb were treated with 1 μM MDI1228, dexamethasone or tofacitinib for 5 days, and then supernatants were collected for analysis. **(H)** ELISA analysis of the expression levels of indicated proteins in T cell supernatants (n=3/group). All error bars indicate mean ± SEM. *p < 0.05, **p < 0.01, ***p < 0.001, ****p < 0.0001.

While T cells drive the pathogenesis of both ACD and AD, the underlying mechanisms differ. ACD is primarily mediated by CD8^+^ cytotoxic T (Tc) cells (6, 9). In the ACD model, MDI1228 but not hydrocortisone significantly reduced the infiltration of CD8^+^ T cells, STAT1 phosphorylation, IFNγ secretion, and the expression of *Ifng*, *Il4*, and *Il17a* (**Figures 3A–C**). Recent studies have defined ACD as a mixed inflammatory condition involving Th1, Th2 and Th17 responses (1, 6, 26). Consistently, MDI1228, as a pan JAK inhibitor, exhibited broad spectrum anti-inflammatory activity by suppressing multiple cytokine pathways. Additionally, MDI1228 treatment reduced the expression of the natural killer (NK) cell marker *Gzma* (27), a finding of particular relevance as NK cells have also been identified as a major source of IFNγ in ACD (6, 28).

In contrast, AD is primarily triggered by Th2 and mast cell activation (29). Both MDI1228 and hydrocortisone significantly suppressed the infiltration of CD4*^+^* T cells, STAT6 phosphorylation, and IL4/IL13 secretion in AD skin, as shown by immunofluorescence staining (**Figures 3D, E**; **Supplementary Figures 3A**, **B**) and qRT-PCR analyses (**Figure 3F**). MDI1228 also downregulated the expression of *Mcpt8*, the marker of basophils, which are also critical producers of IL4 and IL13 in both AD and ACD (6, 30, 31).

To determine if MDI1228 acts directly on T cells, we assessed its effects on T cell differentiation and activation *in vitro* (**Figure 3G**). ELISA and flow cytometry demonstrated that in mouse T cells, MDI1228 suppressed the differentiation of CD8a^+^IFNγ^+^ Tc1 and CD4^+^IL4/IL13^+^ Th2 cells as well as the production of IFNγ, IL4, and IL13 with comparable efficacy to tofacitinib, and both JAK inhibitors were more effective than dexamethasone (**Figure 3H**; **Supplementary Figures 3C**–**E**). Similar effects were observed in human T cells (**Supplementary Figures 3F, G**). Moreover, both MDI1228 and tofacitinib inhibited the expression of the proliferation marker *MKI67* in human T cells (**Supplementary Figure 3G**).

In summary, these findings demonstrate that MDI1228 alleviates type I/II inflammation by directly impeding the activation, differentiation, and cytokine production of key pathogenic T cell subsets.

### 3.4 Transcriptomic profiling identifies dFB-derived CXCL9/10 and CCL2 as potential targets of MDI1228 in ACD and AD

**Figure 4:**
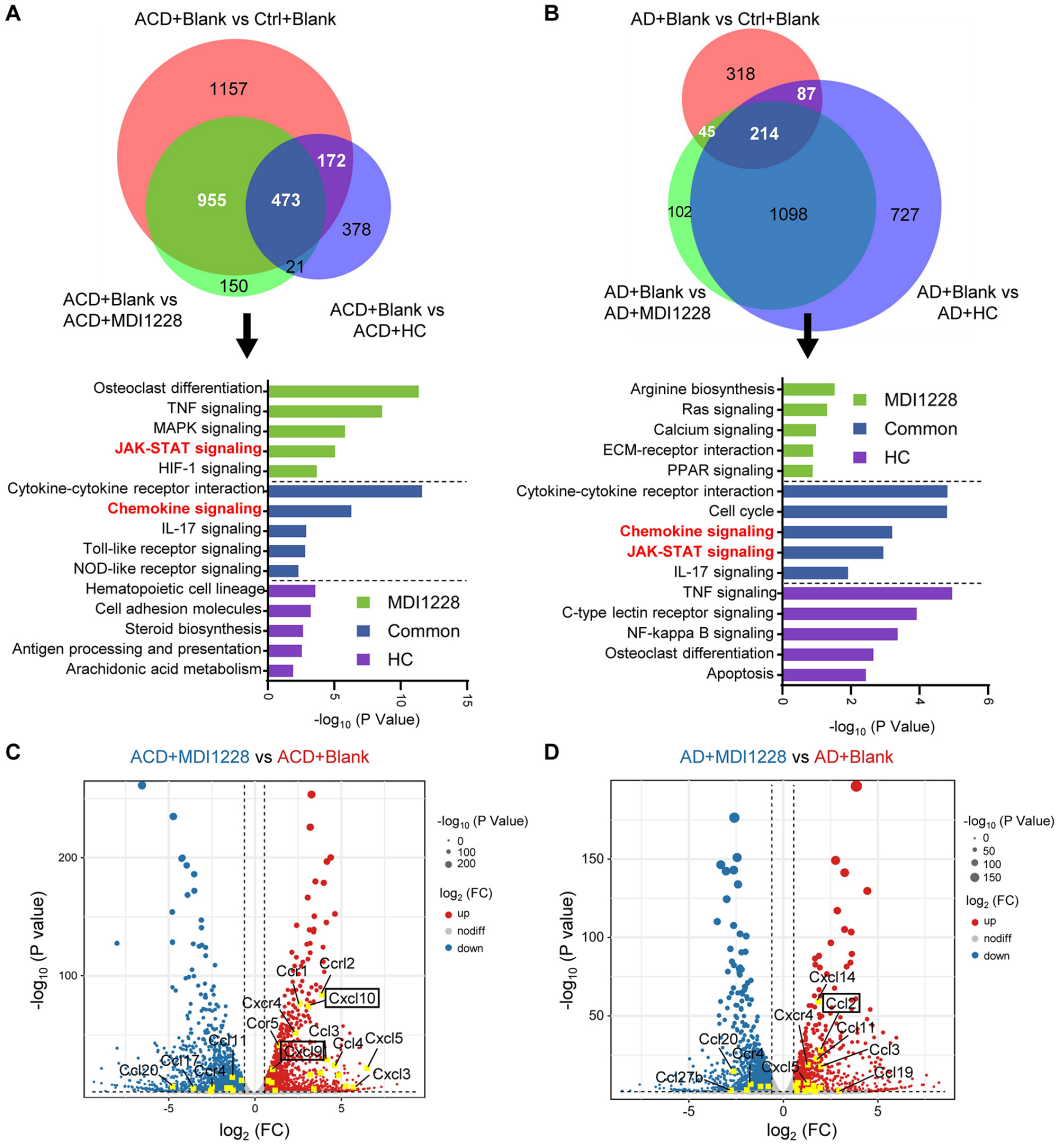
Transcriptomic profiling reveals molecular targets of MDI1228. **(A, B)** Venn diagram and KEGG pathway enrichment of down-regulated genes in the blank, MDI1228, or hydrocortisone-treated skin samples compared to the ACD **(A)** or AD **(B)** model skin samples analyzed by bulk RNA-seq. **(C, D)** Volcano plot of differentially expressed genes between MDI1228-treated group and ACD **(C)** or AD **(D)** group. Red dots indicate genes significantly upregulated in the ACD/AD group and blue dots indicate genes significantly downregulated in the ACD/AD group. The chemokine signatures are highlighted in yellow. Genes with |fold change| > 1.5 and p value < 0.05 are considered significant.

To comprehensively investigate differences in the potential targets of MDI1228 and hydrocortisone, we performed bulk RNA-seq on lesional or non-lesional skin samples from mouse ACD and AD models, followed by KEGG pathway analysis of genes downregulated by each treatment. In ACD, MDI1228 treatment specifically downregulated genes associated with the JAK-STAT pathway, whereas hydrocortisone preferentially inhibited keratinocyte markers (e.g., *Krt16*, *Defb3*, *Defb4*) and antigen presentation genes (e.g., *H2-Aa*, *H2-Ab1*; **Figure 4A**; **Supplementary Figure 4A**). In AD, a substantial overlap of downregulated genes was observed between the two treatments (1,098 out of 2,591 genes; **Figure 4B**). Both MDI1228 and hydrocortisone suppressed JAK-STAT pathway-related genes. IL17 signaling pathway was also enriched among genes downregulated by both MDI1228 and hydrocortisone, including *Il6, Cxcl5, Mmp3 and Mmp13*. Although IL17 has also been reported to participate in AD disease pathogenesis (32–34), these pleiotropic genes are regulated by multiple inflammatory cascades, and their downregulation likely reflects broad-spectrum anti-inflammatory activity rather than specific Th17 inhibition. Moreover, MDI1228 specifically downregulated inflammation-associated fibroblast markers (e.g., *Saa3*, *Prg4*, *Fabp3*) (35–38), while hydrocortisone exhibited broader inhibition of *Tnc*, *Tslp*, *Krt16*, and *Krt17*, all of which are associated with epidermal keratinocyte activation and Th2 inflammation in AD (**Supplementary Figure 4B**) (39–42).

Beyond these distinct effects, both MDI1228 and hydrocortisone commonly suppressed genes involved in cytokine-cytokine receptor interaction and chemokine signaling pathways. Previous reports indicate that in ACD, IFNγ potently induces CXCL9/10/11 expression, which not only recruit type I T cells to inflamed tissues but also promotes naive T cell polarization toward a type I phenotype (6, 43). Conversely, in AD, CCL11, CCL17, and CCL22 are well-characterized Th2-type chemokines (44), and fibroblasts, keratinocytes, as well as various immune cells have been implicated in CCL2-mediated recruitment of Th2 cells and amplification of type II inflammation (45, 46). Notably, CCL2, CCL17, and CCL22 have each been reported to possess the capacity to directly induce Th2 generation or polarization (47, 48).

**Figure 5:**
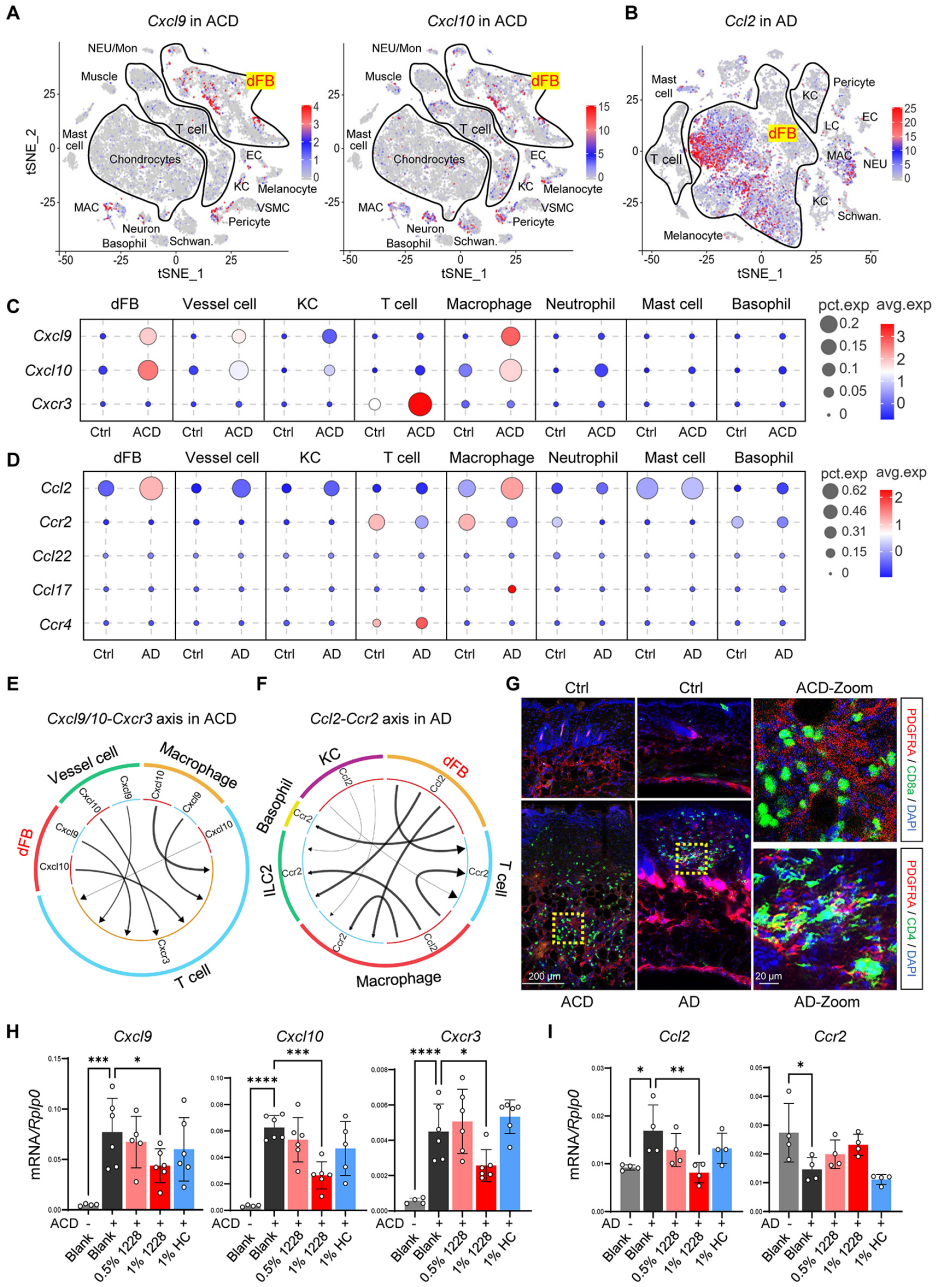
Single−cell transcriptomic analysis reveals the dFB−T cell interactions via chemokine axes in ACD and AD. **(A, B)** tSNE plots showing cell distribution or the expression of *Cxcl9/Cxcl10* in ACD skin **(A)** or *Ccl2* in AD skin **(B)**. **(C, D)** Bubble plots showing the expression of indicated genes for each cell cluster in ACD **(C)** or AD **(D)** skin. **(E, F)** Circle plot from cell-chat analysis showing the inferred intercellular communication network for *Cxcl9/Cxcl10-Cxcr3* signaling in the ACD skin **(E)** and *Ccl2-Ccr2* signaling in the AD skin **(F)**. Only interactions with probability > 1×10^-5^ and p value < 0.05 are shown. Line thickness indicates ligand expression level. The size of the dot at the line terminus indicates receptor expression level. **(G)** Immunostaining of the ACD or AD skin sections with anti-PDGFRA (red) and anti-CD8/CD4 (green) antibodies, and nuclei were counter stained by DAPI (blue). Zoom-in images were shown in the right panel (n=4/group). **(H, I)** qRT-PCR analysis of the mRNA expression levels of indicated genes in ACD **(H)** or AD skin (I, ratios to HK gene *Rplp0* were shown, n=4~6/group). All error bars indicate mean ± SEM. *p < 0.05, **p < 0.01, ***p < 0.001, ****p < 0.0001. *dFB*, dermal fibroblast; *MAC*, macrophage; *NEU*, neutrophil; *Mon*, monocytes; *VSMC*, vascular smooth muscle cell; *KC*, keratinocyte; *EC*, endothelial cell; *Schwan.*, Schwann cell.

Volcano plots showed that MDI1228 treatment significantly inhibited CXCL9/10 in ACD and CCL2 in AD (**Figures 4C, D**). Single-cell transcriptomic analysis identified dFBs and macrophages as the principal cellular sources of CXCL9/10 in ACD and of CCL2 in AD (**Figures 5A–D**). In contrast, CCL17 and CCL22 were barely expressed in either condition and were not the major chemokines suppressed by MDI1228 in AD (**Figures 4D**, **5D**). Intercellular interaction analysis of individual gene sets confirmed that dFBs represent the primary cell cluster that sends chemotactic signals to T cells via the “CXCL” axis in ACD and the “CCL” axis in AD (**Supplementary Figures 4C, D**). Moreover, CellChat analysis and immunofluorescence staining further demonstrated that dFBs interact with T cells through the *Cxcl9/10-Cxcr3* axis in ACD or the *Ccl2-Ccr2* axis in AD (**Figures 5E, F**), with T cell infiltration observed around dFBs (**Figure 5G**). These findings suggest that, beyond conventional keratinocytes or immune cells such as macrophages, dFBs also contribute critically to T cell recruitment into skin lesions. Importantly, MDI1228 treatment reduced the transcriptional expression of *Cxcl9/10* and *Cxcr3* in ACD, as well as *Ccl2* and *Ccr2* in AD (**Figures 5H, I**).

Collectively, our transcriptomic analyses demonstrate that MDI1228 suppresses the production of CXCL9/10 in ACD and CCL2 in AD, thereby blocking dFB-T cell crosstalk in skin lesions. These findings highlight dFB-derived chemokine signaling as a potential therapeutic target for MDI1228 in both ACD and AD.

### 3.5 Single-cell dissection identifies distinct chemokine-producing fibroblast subsets in ACD and AD

**Figure 6:**
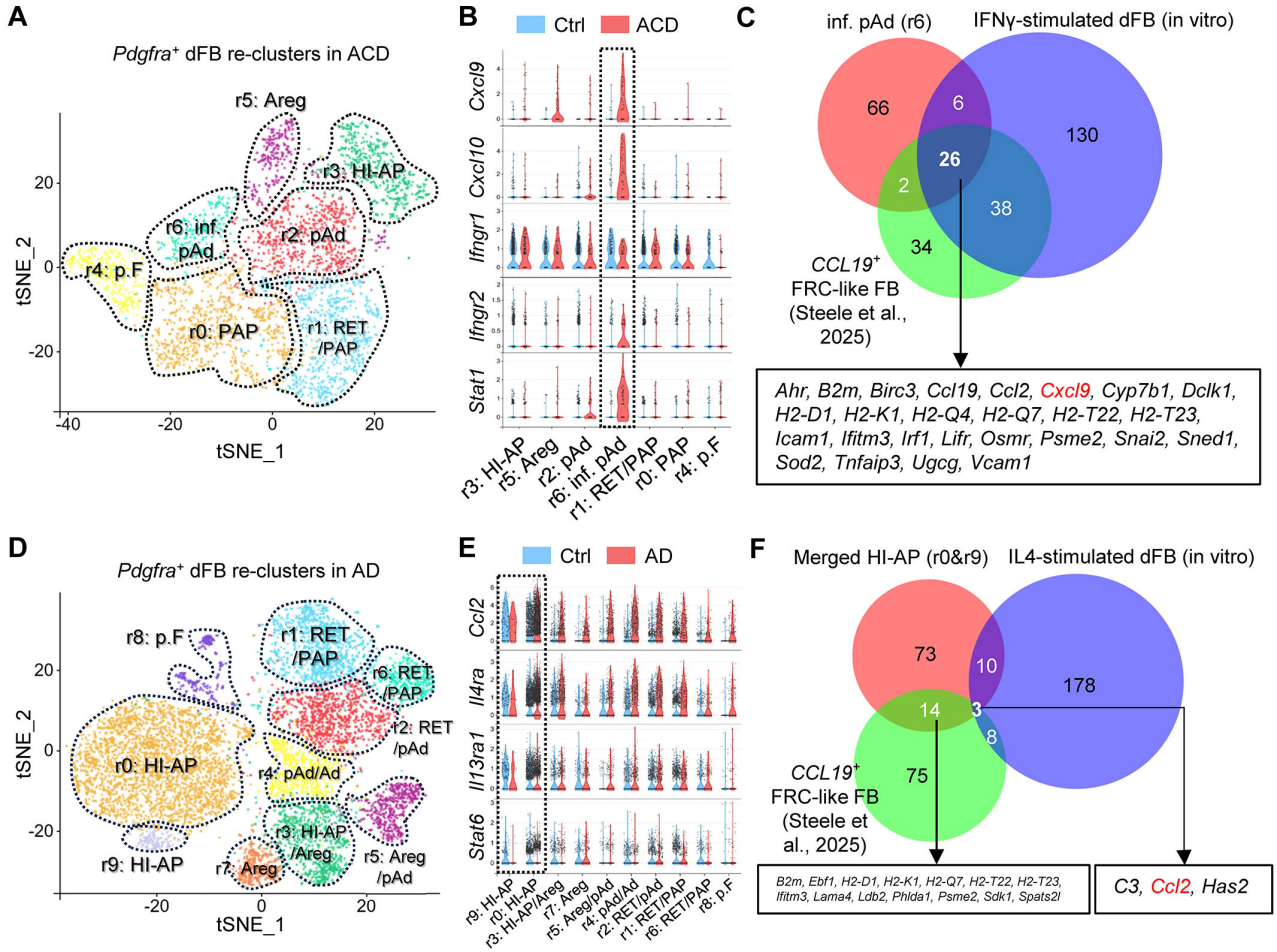
*Pdgfra^+^* dermal fibroblast reclustering reveals the primary sources of pathogenic chemokines in ACD and AD. **(A)** tSNE plots showing cell distribution of the *Pdgfra^+^* dermal fibroblasts after re-clustering in ACD. **(B)** Violin plots showing the expression of indicated genes across various dFB sub-populations in control and ACD samples. **(C)** Venn diagram comparing the top 100 differential expressed genes upregulated in *CCL19*^+^*CD74*^+^*HLA-DRA*^+^fibroblastic reticular cell-like fibroblasts (Steele et al., 2025), the top 100 genes enriched in r6: inflammatory pre-adipocytes in ACD, and the top 200 IFNγ-inducible genes in primary dFBs (*in vitro*). **(D)** tSNE plots showing cell distribution of the *Pdgfra^+^* dermal fibroblasts after re-clustering in AD. **(E)** Violin plots showing the expression of indicated genes across various dFB sub-populations in control and AD samples. **(F)** Venn diagram comparing the differential expressed genes upregulated in *CCL19*^+^*CD74*^+^*HLA-DRA*^+^fibroblastic reticular cell-like fibroblasts (Steele et al., 2025), the top 100 genes enriched in merged HI-AP cluster of r0 and r9 in AD, and the top 200 IL4-inducible genes in primary dFBs (*in vitro*). *HI-AP*, hypodermal interstitium adipocyte progenitor; *Areg*, adipogenesis-regulatory cell; *pAd*, pre-adipocyte; *inf. pAd*, inflammatory pre-adipocyte; *RET/PAP*, reticular and/or papillary dFB; *p.F*, peri-follicular dFB.

Recent studies have established that dFBs are highly heterogeneous and comprise functionally distinct subsets in inflamed skin (45, 49, 50). To define the specific dFB subpopulations responsible for pathogenic chemokine production in ACD and AD, we performed unbiased sub-clustering of *Pdgfra^+^* dFBs in ACD and AD. Based on previously established dFB and adipocyte-lineage marker gene profiles (51–56), the dFB sub-clusters in ACD were defined as *Trps1^+^Lrig1^+^* papillary dFBs (PAP, r0), *Cyp4b1^+^Lrig1^hi^* reticular or papillary dFBs (RET/PAP, r1), *Ly6a^+^Icam1^+^* pre-adipocytes (pAd, r2), *Mfap5^+^Col14a1^+^* hypodermal interstitium adipocyte progenitors (HI-AP, r3), *Prdm1^hi^Trps1^hi^* peri-follicular dFBs (p.F, r4), *Fmo2^+^Mgp^+^* adipose regulatory cells (Areg, r5), and *Ly6a^+^Icam1^+^Ccl19^+^* inflammatory pre-adipocytes (inf. pAd, r6; **Figure 6A**; **Supplementary Figures 5A, B**). Among these, inf. pAd-r6 exhibited enrichment of *Cxcl9 and Cxcl10*, *Ifngr2*, and *Stat1* expression (**Figure 6B**; **Supplementary Figure 5C**). Steele et al. recently identified a conserved disease-adapted CCL19^+^CD74^+^HLA-DRA^+^ fibroblastic reticular cell (FRC)-like fibroblast subtype in human skin that highly expressed *CXCL9* during tissue injury and inflammation (49). Notably, inf. pAd-r6 in ACD similarly exhibited high expression of *Ccl19, Cd74*, and MHC-I-related genes such as *H2-K1* (**Supplementary Figures 5A, 5D**). Comparison of differentially upregulated genes between human CCL19^+^ FRC-like fibroblasts and inf. pAd-r6 in ACD revealed that both cell types shared similar IFNγ-downstream gene expression (e.g. *Cxcl9, Irf1, Ifitm3, and Psme2*) (57–59), as well as antigen presentation pathway-related genes (e.g. *B2m, H2-D1, H2-K1*, and *H2-Q4*; **Figure 6C**) (60). These findings defined inf. pAd-r6 as the principal dFB source of CXCL9/10 in ACD and identify this subset as the murine counterpart of a conserved pathogenic fibroblast population in human inflammatory skin diseases.

Similarly, we performed dFBs re-clustering in AD and identified multiple subsets, including *Mfap5^+^Col14a1^+^* HI-APs (r0/r9), *Cyp4b1^+^Lrig1^+^* RET/PAPs (r1/r6), *Cyp4b1^+^Icam1^+^* RET/pAds (r2), *Mfap5^+^Mgp^+^* HI-AP/Aregs (r3), *Icam1^+^Lpl1^hi^Pparg^hi^* pAd/Ads (r4), *Mgp^+^Apoe^+^* Areg/pAds (r5), *Fmo2^+^Mgp^+^* Aregs (r7), and *Prdm1^hi^Trps1^hi^* peri-follicular dFBs (r8; **Figure 6D**; **Supplementary Figures 5E, F**). *Ccl2*, *Il4ra*, *Il13ra1*, and *Stat6* transcripts were enriched in HI-AP-r0 (**Figure 6E**; **Supplementary Figure 5G**). In human AD skin, COL6A5^+^COL18A1^+^ fibroblasts have been reported as major producers of CCL2 (45). Interestingly, in mouse AD, only HI-AP-r9 exhibited a *Col6a5^+^Col18a1^+^* transcriptional signature (**Supplementary Figure 5H**). We therefore merged HI-AP-r0 and r9 and compared their differentially upregulated genes with those of CCL19^+^ FRC-like FBs. This analysis revealed that only a limited set of IL4-inducible genes, including *Ccl2*, were commonly upregulated in both FB populations, while both subsets also shared high expression of antigen presentation genes (e.g. *B2m, H2-D1, H2-K1, H2-Q7, H2-T22*, and *H2-T23*; **Figure 6F**). These observations suggest that the pathogenic phenotype of key fibroblast subsets in mouse and human AD may depend on additional pro-inflammatory signals beyond IL4.

Together, our single-cell dissection identifies an IFNγ-responsive *Cxcl9/10^+^* inflammatory preadipocyte subset in ACD and an IL4-responsive *Ccl2^+^* HI-AP population in AD as the principal dFB sources of key T cell chemokines, both of which mirror conserved pathogenic fibroblast subsets reported in human inflammatory skin diseases.

### 3.6 MDI1228 suppresses dermal fibroblast-derived chemokines to block pathogenic T cell polarization

**Figure 7:**
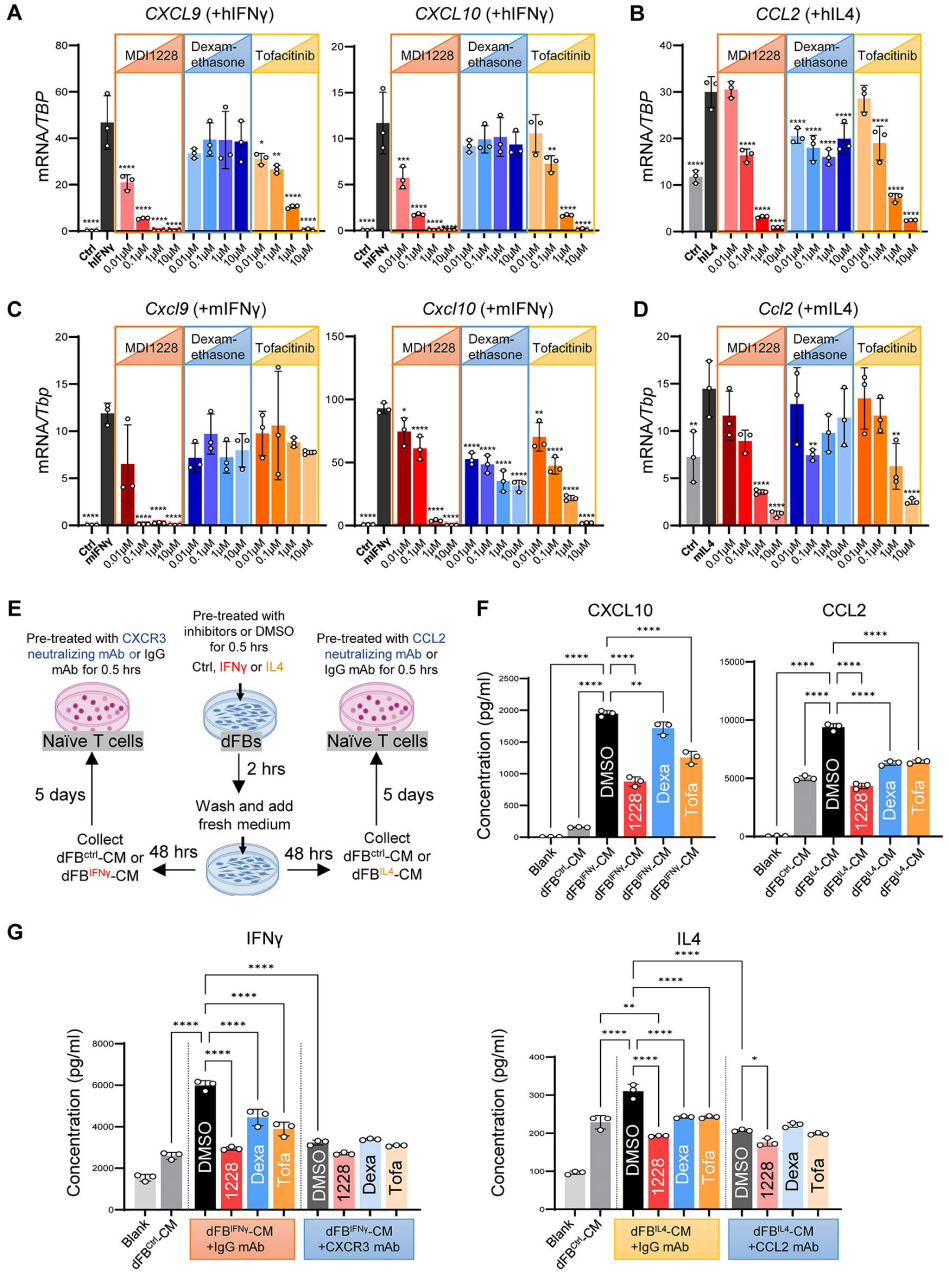
MDI1228 disrupts fibroblast-T cell crosstalk via chemokine signaling *in vitro.* **(A, B)** Human dFBs were pre-treated with MDI1228, tofacitinib or dexamethasone (0.01 μM, 0.1 μM, 1 μM, or 10 μM) for 30 min and then treated with hIFNγ **(A)** or hIL4 **(B)** for 24 hrs. Control or cytokine-treated samples were subjected to qRT-PCR analysis of indicated gene mRNA expression (n=3/group). **(C, D)** Mouse dFBs were pre-treated with MDI1228tofacitinib or dexamethasone (0.01 μM, 0.1 μM, 1 μM, or 10 μM) for 30 mins and then treated with mIFNγ **(C)** or mIL4 **(D)** for 24 hrs. Control or cytokine-treated samples were subjected to qRT-PCR analysis of indicated gene mRNA expression (n=3/group). **(E)** Experiment scheme for collection of IFNγ or IL4-primed dFB conditioned medium (dFB^IFNγ^-CM or dFB^IL4^-CM) or control dFB^ctrl^-CM to stimulate naïve T cells. Primary dFBs were pre-treated with 1% DMSO, MDI1228, dexamethasone and tofacitinib for 30 mins and then treated with IFNγ or PBS control for 2 hrs. Cells were washed twice with PBS then replenished with fresh medium without IFNγ or IL4 for additional 48 hrs, and CM was collected for the 5-day-co-culture with T cells pre-treated by CXCR3 or CCL2 neutralizing antibody for 30 mins. **(F)** ELISA analysis of CXCL10 or CCL11 expression levels in dFB^ctrl^-CM, dFB^IFNγ^-CM or dFB^IL4^-CM (n=3/group). **(G)** Naïve T lymphocytes pre-treated with CXCR3 or CCL2 neutralizing antibody were stimulated with dFB^ctrl^-CM, dFB^IFNγ^-CM or dFB^IL4^-CM, and cell supernatants were collected for ELISA analysis of IFNγ or IL4 protein expression (n=3/group). All error bars indicate mean ± SEM. *p < 0.05, **p < 0.01, ***p < 0.001, ****p < 0.0001.

To determine whether MDI1228 inhibits type I or type II cytokine-induced chemokine expression, we treated human or mouse primary dFBs with 0.1-10 μM MDI1228, dexamethasone, or tofacitinib following IFNγ or IL4 stimulation. At the transcriptional level, MDI1228 more effectively inhibited IFNγ-induced *CXCL9* and *CXCL10* expression in human dFBs than tofacitinib or dexamethasone, particularly at lower concentrations (0.01 µM and 0.1 µM; **Figure 7A**). However, no significant differences between MDI1228 and tofacitinib were observed in suppressing IL4-induced *CCL2* expression (**Figure 7B**). In mouse dFBs, 1 μM MDI1228 suppressed IFNγ-induced *Cxcl9* and *Cxcl10* expression by over 90%, respectively. In contrast, only10 μM tofacitinib achieved over 90% inhibition of *Cxcl10* but showed no obvious inhibition of *Cxcl9* (**Figure 7C**). The two drugs exhibited comparable inhibition of IL4-induced *Ccl2* expression (**Figure 7D**). The differential drug potency between mouse and human cells may be attributable to species variations in JAK protein sequences and conformational changes (15).

Next, to determine if MDI1228 impairs T cell pro-inflammatory polarization by inhibiting dFB-derived chemokines, we collected conditioned medium from mouse dFBs pre-treated with drugs and stimulated with IFNγ or IL4 (dFB^IFNγ^-CM or dFB^IL4^-CM) for co-culture with naïve T cells that had been pre-incubated with neutralizing antibodies against CXCR3 or CCL2 (**Figure 7E**). ELISA analysis showed that MDI1228 uniquely reduced both CXCL10 in dFB^IFNγ^-CM and CCL2 in dFB^IL4^-CM (**Figure 7F**), and these CMs contained no detectable IFNγ or IL4 that could interfere with T cell differentiation (**Supplementary Figure 6A**). Subsequently, compared with dFB^Ctrl^-CM, dFB^IFNγ^-CM and dFB^IL4^-CM significantly enhanced T cell production of IFNγ and IL4, respectively, and these effects were effectively blocked by pre-treating dFBs with MDI1228 or pre-treating T cells with neutralizing antibodies against CXCR3 or CCL2 (**Figure 7G**). In co-culture assays using human dFB-derived CM, hdFB^IFNγ^-CM similarly promoted the expression of *TBX21* (a key type I T cell transcription factor) and *IFNγ*, whereas dFB^IL4^-CM failed to induce consistent Th2 activation (**Supplementary Figures 6B, C**), likely due to the high basal expression of CCL2 in unstimulated human dFBs.

Delgocitinib is an established topical pan-JAK inhibitor approved for the treatment of atopic dermatitis and chronic hand eczema (61, 62). We therefore compared the anti-inflammatory activity of MDI1228 and delgocitinib in mouse dFBs. qRT-PCR analysis showed that at the same concentration, MDI1228 was more effective than delgocitinib in suppressing IFNγ-induced *Cxcl9* and *Cxcl10.* For example, at 1 μM, MDI1228 achieved 93.8% inhibition of *Cxcl9* compared to 63.8% for delgocitinib, whereas both drugs comparably inhibited IL4-induced *Ccl2* (**Supplementary Figures 6D, E**). Consistent with these transcriptional data, ELISA of drug-treated dFB-CM revealed that MDI1228 reduced CXCL10 in dFB^IFNγ^-CM to a slightly lower level than delgocitinib, while CCL2 levels in dFB^IL4^-CM were comparable between the two drugs (**Supplementary Figures 6F, G**). Functionally, the co-culture assays further demonstrated that both MDI1228 and delgocitinib blocked T cell production of IFNγ or IL4 through the CXCR3 and CCL2 pathways (**Supplementary Figure 6H**). To further exclude the possibility that residual drug carryover could directly influence T cells, we also performed a drug-only wash control. Cell-free wells were supplemented with MDI1228 or delgocitinib and subjected to the same washing and medium-exchange procedure (**Supplementary Figure 6F**). The resulting control media did not affect T cell production of IFNγ or IL4 (**Supplementary Figure 6H**), confirming that the observed effects were mediated by dFB-conditioned medium rather than residual drug carryover.

These results demonstrate that beyond directly targeting T cells, MDI1228 inhibits IFNγ-induced CXCL10 secretion in ACD and IL4-induced CCL2 secretion in AD from dFBs, thereby blocking the polarization of pathogenic T cells.

### 3.7 Repeated topical MDI1228 administration for 14 days exhibits minimal systemic adverse effects

**Figure 8:**
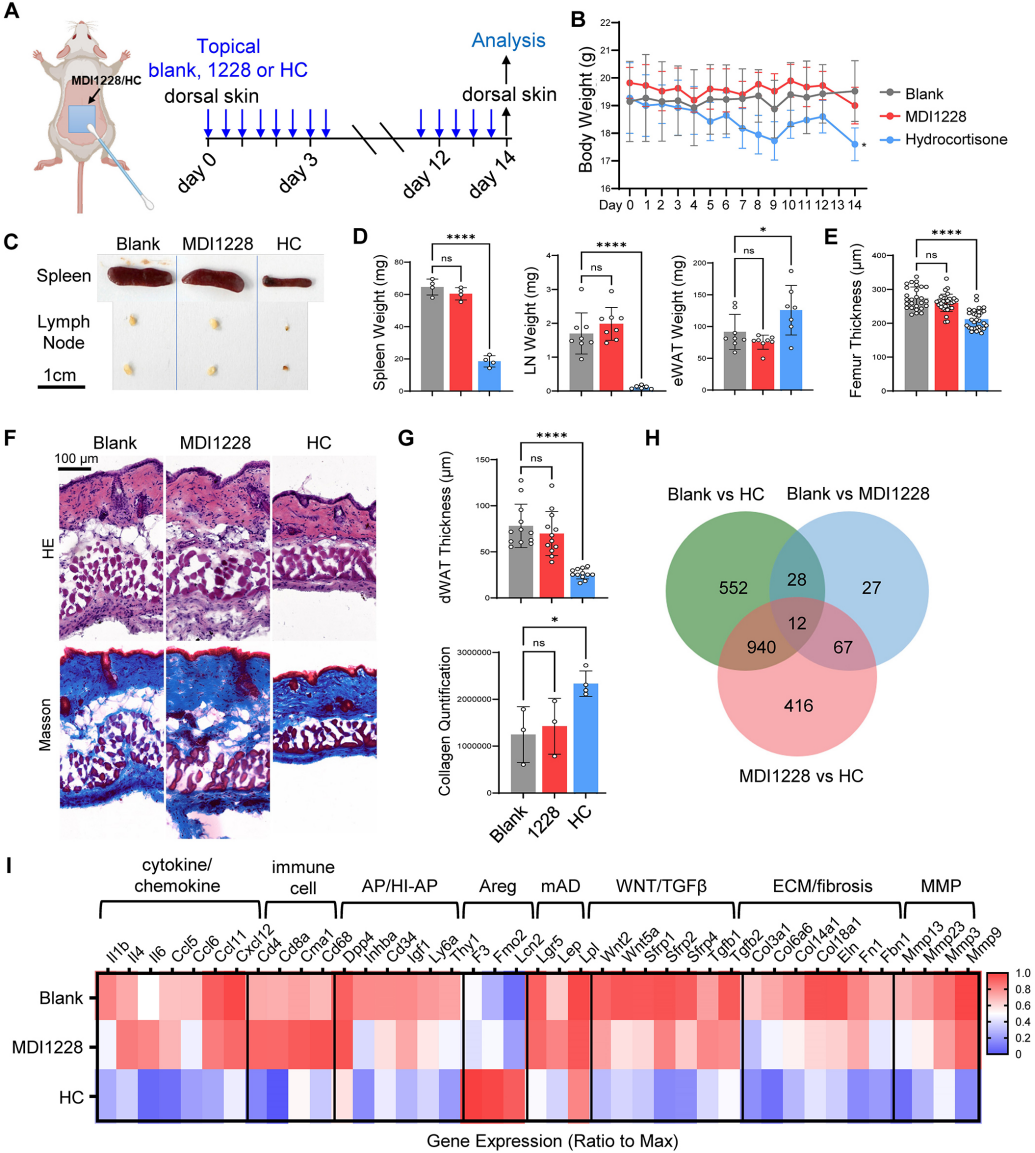
Repeated topical MDI1228 administration for 14 days demonstrates a favorable safety profile. **(A)** Overview of the experimental setting. 1% MDI1228, hydrocortisone or blank hydrogel was topically applied on dorsal skin twice a day for 2 weeks. Dorsal skin and immune organs were collected at day 14 for analyses. **(B)** Body weight of the mice for each group from day 0 to day 14 (n=4/group). Comparisons were performed between blank group and each other group via two-way analysis of variance. **(C-E)** Representative images **(C)**, quantified results showing weight **(D)** of spleens, lymph nodes or eWATs and femur thickness **(E)** for each group at day 14 (n=4, 8 or 36/group). **(F-G)** H&E staining and masson staining **(F)** of skin sections for each group at day 14. Quantified results **(G)** showing the dWAT layer thickness (n=12/group) and the collagen expression (n=3~4/group) mouse skin. **(H)** Mouse skin for each group was subjected to RNA-seq analysis. Venn diagram comparing differential genes in Ctrl vs HC, Ctrl vs 1228 or MDI1228 vs HC groups. **(I)** Heat map of indicated gene expressions in mouse skin for each group by RNA-seq analysis. All error bars indicate mean ± SEM. *p < 0.05, **p < 0.01, ***p < 0.001, ****p < 0.0001.

Prolonged use of topical glucocorticoids is associated with local and systemic adverse effects, including skin atrophy, telangiectasia, and immunosuppression (63). Although long-term safety data for topical JAK inhibitors are limited, current evidence indicates a relatively favorable safety profile (64). To evaluate the systemic safety of MDI1228, we applied 1% MDI1228 hydrogel or hydrocortisone ointment topically to mouse dorsal skin for 14 consecutive days (**Figure 8A**). Phenotypic analysis revealed that hydrocortisone induced systemic toxicity, evidenced by reduced weight of spleen and lymph nodes, thinning of leg bones, dWAT atrophy, and excessive collagen deposition (**Figures 8B–G**). In contrast, MDI1228 treatment did not produce these adverse effects under the same regimen. Transcriptomic and qRT-PCR analyses further showed that hydrocortisone significantly downregulated genes involved in immune responses, adipocyte differentiation, WNT/TGFβ signaling, and ECM/fibrosis, whereas repeated MDI1228 application showed minimal impact on these pathways (**Figures 8H, I**; **Supplementary Figure 7A**).

Collectively, these results indicate that topical application of MDI1228 for 14 consecutive days does not elicit systemic immunosuppression or tissue atrophy, supporting its favorable safety profile.

## 4 Discussion

In this study, we developed MDI1228, a novel topical pan-JAK inhibitor, and demonstrated its therapeutic efficacy in both ACD-like and AD-like dermatitis mouse models. Mechanistically, MDI1228 exerts dual actions by directly suppressing pathogenic T cell differentiation and activation and by indirectly disrupting dFB-T cell crosstalk through the inhibition of dFB-derived chemokine production, specifically CXCL9/10 in the Tc1 -driven ACD and CCL2 in the Th2-driven AD. MDI1228 also exhibited a favorable local safety profile with minimal systemic adverse effects after 2-week topical administration, although direct evidence for a ‘soft drug’ mechanism is currently lacking. These findings position dFB-derived chemokine axes as a therapeutic target across these two distinct autoimmune skin inflammations and highlight the potential of MDI1228 as a safe and effective treatment option.

Traditionally viewed as passive structural components, dFBs are now recognized as dynamic immunomodulators that actively shape the inflammatory microenvironment. Upon activation by IFNγ or IL4/IL13, dFBs secrete chemokines and cytokines that recruit and retain pathogenic immune cells, amplifying inflammation in psoriasis, vitiligo, and lupus (5–7, 10, 35, 50, 65–67). Our findings demonstrate that MDI1228 potently suppresses JAK-STAT signaling in dFBs, inhibiting the production of CXCL10 in IFNγ-stimulated cells and CCL2 in IL4-stimulated cells. This dual blockade is particularly significant given that single-cell studies identify dFBs as the primary source of these chemokines in inflammatory skin diseases (5, 6, 50). These findings have led to the proposal that targeting aberrantly activated dFBs could be a promising therapeutic strategy for treating inflammatory and autoimmune dermatitis.

A key finding of our study is the role of disease-specific chemokine pathways in promoting T-cell polarization. The CXCL9/CXCL10-CXCR3 axis critically regulates Type I immune recruitment and activation (68–71). In ACD, IFNγ-activated dFBs drive CD8^+^ Tc1 polarization via CXCL10, creating positive feedback loops that amplify tissue damage (6), which aligns with reports implicating this axis in vitiligo and contact dermatitis pathogenesis (50, 69). In contrast, the chemokine landscape in AD is distinct, with CCL2 emerging as a dominant mediator in our model. CCL2 has been shown to directly induce Th2 polarization of CD4^+^ T cells, promoting the secretion of IL2, IL4 and IL5 while inhibiting IFNγ production (72, 73). However, accumulating evidence indicates that CCL2 can also drive Th1 and CD8^+^ T cell responses under other inflammatory conditions. Neutralization of CCL2 or genetic ablation of its receptor CCR2 has been shown to impair Th1 immunity in models of viral myocarditis and pulmonary fungal infection (74, 75). Additionally, during viral infection, CCL2 is required for CD8^+^ T cell recruitment and activation (76). These observations highlight how the function of a single chemokine can be reshaped by the prevailing cytokine environment, emphasizing the importance of targeting disease-specific chemokine axes therapeutically.

It is important to note that MDI1228 is a non-selective pan-JAK inhibitor. It’s *in vivo* efficacy likely reflects combined effects on multiple skin-resident cells. In addition to dFBs, JAK inhibition also attenuates chemokine production in keratinocytes, endothelial cells, and immune cells, which contribute to inflammatory signaling in skin lesions. For instance, ruxolitinib and tofacitinib have been reported to suppress CCL2 and CXCL9/10/11 expression in macrophages from rheumatoid arthritis or IFNβ stimulation (77, 78). Additionally, delgocitinib alleviates AD by inhibiting the production of CCL26 and CXCL6 in IL4/IL13-triggered keratinocytes (79). Thus, the therapeutic effects of MDI1228 may involve coordinated suppression across multiple cell types. Nevertheless, our single-cell analyses consistently identify dFBs as the predominant source of CXCL9, CXCL10, and CCL2 in ACD and AD, supporting a primary role for stromal cells in orchestrating T cell recruitment and polarization. We cannot exclude indirect effects on T cells via macrophages or other antigen-presenting cells, a question that warrants future cell-type-specific deletion studies.

Although MDI1228 is designed as a pan-JAK inhibitor, we cannot exclude potential off-target effects. Several JAK inhibitors are known to interact with kinases beyond the JAK family (80). For instance, tofacitinib has been reported to inhibit TRPM6 and PKN2 in addition to its primary JAK targets (81), while ruxolitinib still inhibits at least 12 kinases at ≥50% activity (82). AZD1480, originally a JAK2 inhibitor, also potently inhibits FGFR3 (83). Thus, off-target FGFR inhibition is not uncommon among JAK-targeted compounds. Although often considered an undesirable liability, concurrent inhibition of FGFR and JAK-STAT may confer benefits in prostate cancer and colorectal cancer (84, 85). These observations raise the possibility that simultaneous FGFR and JAK inhibition is being actively pursued as a rational therapeutic strategy. Nevertheless, the transcriptional profiles identified in this study indicate that topical application of MDI1228 does not significantly modulate the FGFR signaling pathway in mouse skin. Additionally, LTK is unlikely to contribute significantly, as its expression in dFBs is minimal. Therefore, while acknowledging the multi-target nature of MDI1228, our data support JAK pathway inhibition in dFBs as the dominant mechanism in ACD and AD.

Systemic JAK inhibitors, while highly efficacious, carry boxed warnings for serious infections, herpes zoster reactivation, venous thromboembolism, malignancies, and major adverse cardiovascular events (86). In contrast, topical JAK inhibitors such as ruxolitinib and delgocitinib offer targeted therapy with substantially reduced systemic side effects. Clinical trials consistently report primarily mild application-site reactions (e.g., acne, erythema, folliculitis), with systemic events such as upper respiratory tract infections occurring at rates comparable to placebo (87). In our rat toxicokinetic study, topical MDI1228 demonstrated a favorable safety profile. Noticeable drug accumulation occurred only at 120 mg/kg/day (corresponds to a human equivalent topical dose of ~19.4 mg/kg/day), with no drug-related changes in hematological parameters, organ weights, or local skin reactions. For clinical context, 1.5% ruxolitinib applied to ≤20% BSA (~1.84 mg/kg/day) produces a geometric mean steady-state plasma concentration of 14.2 nM (~14.4 ng/mL) (25). Using the FDA-recommended body surface area conversion factor (mouse to human: 12.3) (19), the therapeutic mouse doses of 25–50 mg/kg/day correspond to human equivalent topical doses of ~1.0-4.1 mg/kg/day, at which the systemic exposure of MDI1228 (C_max_=1.04~3.2 nM) is expected to be comparable to that of ruxolitinib (C_max_=3.98 ± 3.5 nM) (87). Critically, marked accumulation occurred only at 120 mg/kg/day, accompanied by a supra−proportional exposure increase from 40 to 120 mg/kg/day (26.6−fold AUC increase for a 3−fold dose increase in males) and a skin/plasma ratio exceeding 1,300−fold, indicating a skin depot−driven mechanism rather than systemic clearance saturation. Thus, the accumulation is a dose−specific phenomenon rather than a general concern for repeated application. Collectively, MDI1228 exhibits a safety profile consistent with that of approved topical JAK inhibitors at clinically relevant doses, combining effective local targeting with no detectable toxicity.

Our study has several limitations. First, our findings are derived primarily from murine ACD and AD models, which may not fully recapitulate human disease. Human cell validation was limited to cultured dFBs and T cells, and clinical evidence for JAK inhibitors in ACD remains sparse compared to AD. Second, while single-cell and spatial analyses support dFB-T cell crosstalk, definitive causal relationships and the *in vivo* contribution of CXCL9/10-CXCR3 and CCL2-CCR2 signaling require cell type-specific genetic approaches or chemokine receptor blockade studies. Third, the specific contribution of individual JAK isoforms to the therapeutic effects of MDI1228 remains to be determined. Fourth, MDI1228 likely acts on multiple JAK-expressing cell types *in vivo*; without conditional knockouts or targeted delivery, we cannot definitively attribute its efficacy solely to T cells and dFBs. Fifth, toxicokinetic evaluation was performed in rats whereas pharmacodynamic efficacy was assessed in mice, creating a species mismatch. Although rats are standard for dermal absorption (88, 89), future studies should quantify MDI1228 concentrations in mouse skin to directly correlate local exposure with efficacy. Sixth, treatment for ACD was initiated 12 hrs. after elicitation. ACD is a classic delayed−type hypersensitivity reaction in which inflammation typically peaks at 48~96 hrs. post−elicitation in both mice and humans (90–92). Thus, our treatment window preceded the peak inflammatory response, making this an early−intervention rather than a true therapeutic model. Future studies should include later treatment initiation of MDI1228 and employ alternative sensitizers to better mimic clinical settings. Finally, our acute inflammation models do not address chronic relapsing disease or potential tachyphylaxis.

## 5 Conclusion

In conclusion, this study identifies dFBs as central regulators of chemokine-mediated T cell polarization in autoimmune dermatitis and demonstrates that selective inhibition of dFB-derived chemokine signaling represents an effective therapeutic strategy. By delineating distinct chemokine axes in ACD (CXCL9/10-CXCR3) and AD (CCL2-CCR2), and revealing their sensitivity to JAK inhibition, we establish MDI1228 as a potent and safe topical pan-JAK inhibitor. Single-cell dissection identified the IFNγ-responsive *Cxcl9/10^+^* inflammatory preadipocytes in ACD and the IL4-responsive *Ccl2^+^* HI-APs in AD as the principal dFB sources of pathogenic chemokines, and these dFB subpopulations are conserved in human inflammatory skin diseases. MDI1228 effectively suppresses JAK-STAT signaling in both T cells and dFBs, thereby disrupting pathogenic dFB-T cell crosstalk while preserving tissue homeostasis and avoiding the adverse effects associated with prolonged glucocorticoid use. The broad applicability of JAK-STAT blockade in fibroblast activation suggests potential utility extending beyond ACD and AD to other autoimmune conditions including psoriasis, lupus erythematosus, and chronic urticaria, where similar cytokine-driven stromal-immune interactions drive pathology.

## Data Availability

The data presented in the study are deposited in the GEO database, accession number GSE224848 and GSE308391.
