# Supplementary material for: MDI1228, a topical pan-JAK inhibitor, disrupts dermal fibroblast-T cell chemokine crosstalk to resolve allergic contact and atopic dermatitis
Source: Front Immunol. 2026 Jul 20;17:1875876. doi: 10.3389/fimmu.2026.1875876 (PMC13429593; doi:10.3389/fimmu.2026.1875876)
Supplement: Supplementary file 1 [file Table1.docx]

Supplementary Material

# Supplementary Figures

**
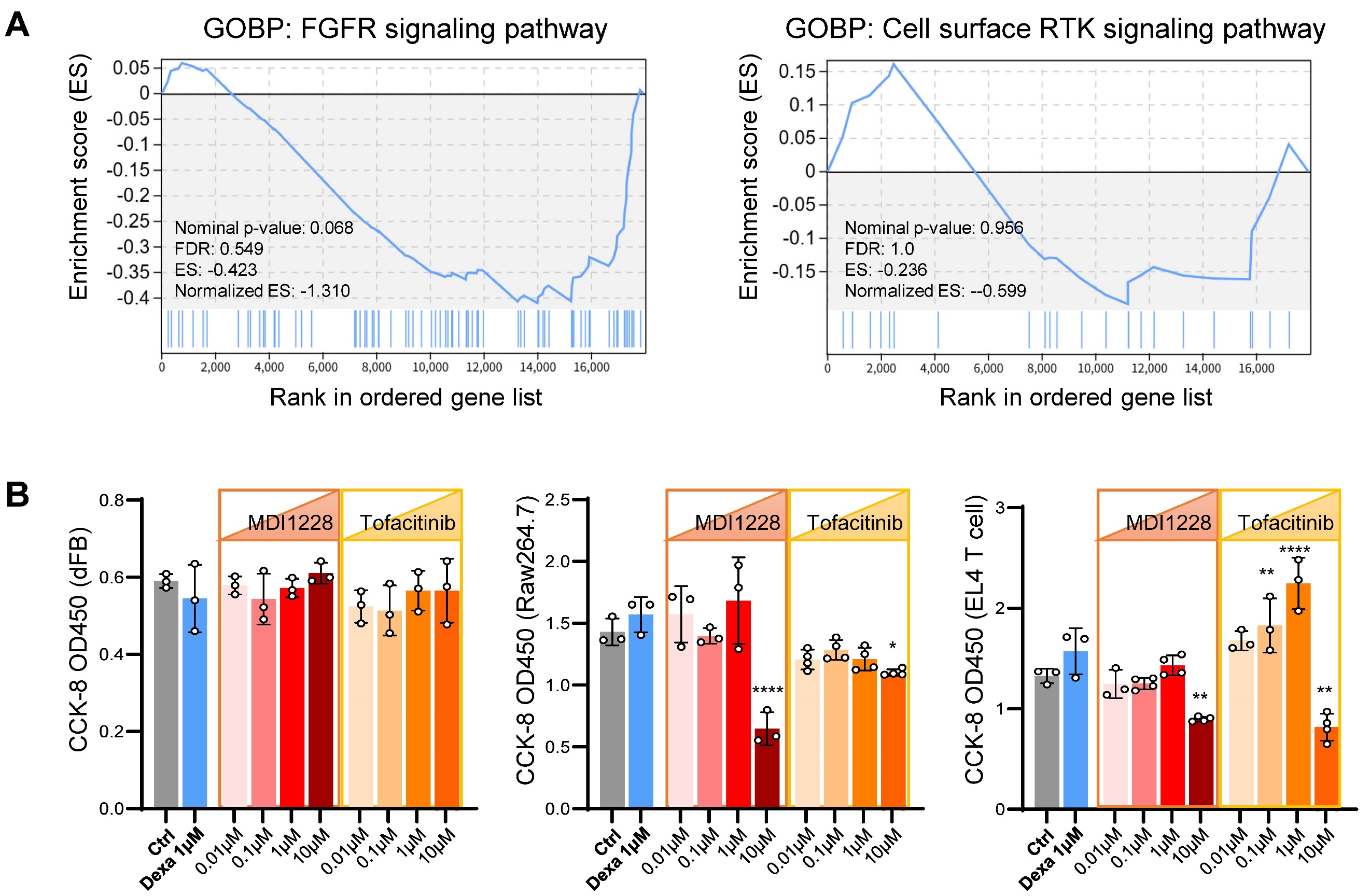
**

**Figure S1. Evaluation of pharmacological activity and in vitro toxicity of MDI1228**

(**A**) Gene Set Enrichment Analysis (GSEA) of GO Biological Process terms reveals activation of FGFR and cell surface RTK signaling pathways by MDI1228 in mouse skin after 14‑day topical treatment. Differentially expressed genes between MDI1228‑treated and blank vehicle‑treated mouse skin (n=4/group) were ranked by signal‑to‑noise ratio.

(**B**) CCK-8 assay of MDI1228 in dFBs, Raw264.7 macrophages or EL4 T cells. dFBs, Raw264.7 or EL4 cells were treated by MDI1228, tofacitinib (0.01 μM, 0.1 μM, 1 μM or 10 μM) or dexamethasone (1 μM) at a cell density of 80%, and OD450 was measured at 24 hrs post-treatment. Comparisons were performed between control and each group via one-way analysis of variance.

All error bars indicate mean ± SEM. *p < 0.05, **p < 0.01, ***p < 0.001, ****p < 0.0001.


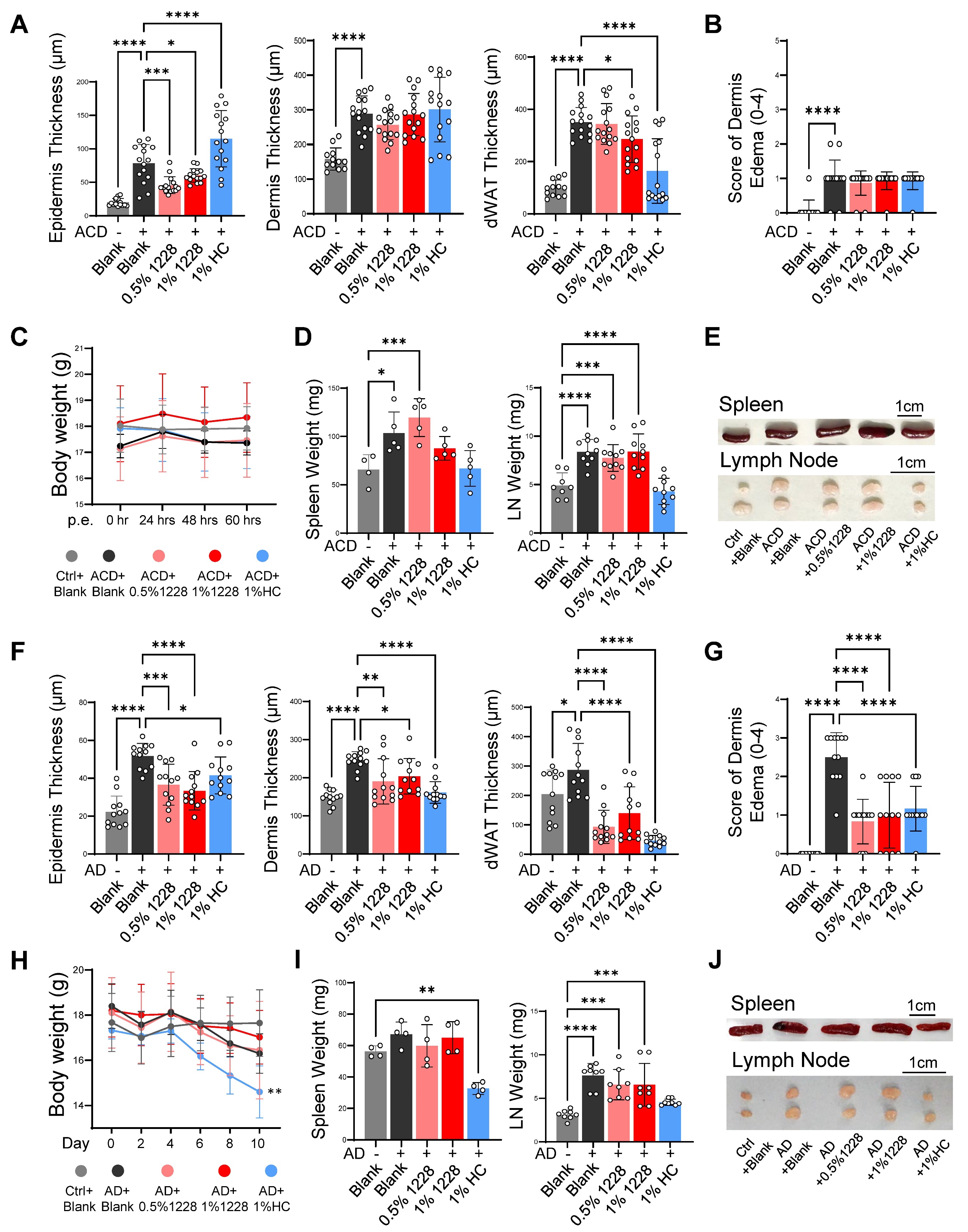


**Figure S2. Systemic and skin histomorphometric parameters of MDI1228 in ACD and AD**

(**A-B**) Quantification of the thickness of epidermis, dermis and dWAT (A) and score of dermis edema (**B**) in ACD skin sections for each group at 60 hrs after elicitation (n=12~15/group).

(**C**) Body weight of the mice for each group from 0 hr to 60 hrs post-elicitation (n=4~5/group).

(**D-E**) Weight (**D**) and representative images (**E**) of mouse spleens and lymph nodes for each group at 60 hrs post-elicitation (n=4~5 or 8~10/group).

(**F-G**) Quantification of the thickness of epidermis, dermis and dWAT (**F**) and score of dermis edema (**G**) in AD skin sections for each group at Day 10 (n=12/group).

(**H**) Body weight of the mice for each group from day 0 to day 10 after MC903 stimulation (n=4/group).

(**I-J**) Weight (**I**) and representative images (**J**) of mouse spleens and lymph nodes for each group at Day 10 (n=4 or 8/group).

All error bars indicate mean ± SEM. *p < 0.05, **p < 0.01, ***p < 0.001, ****p < 0.0001.

**
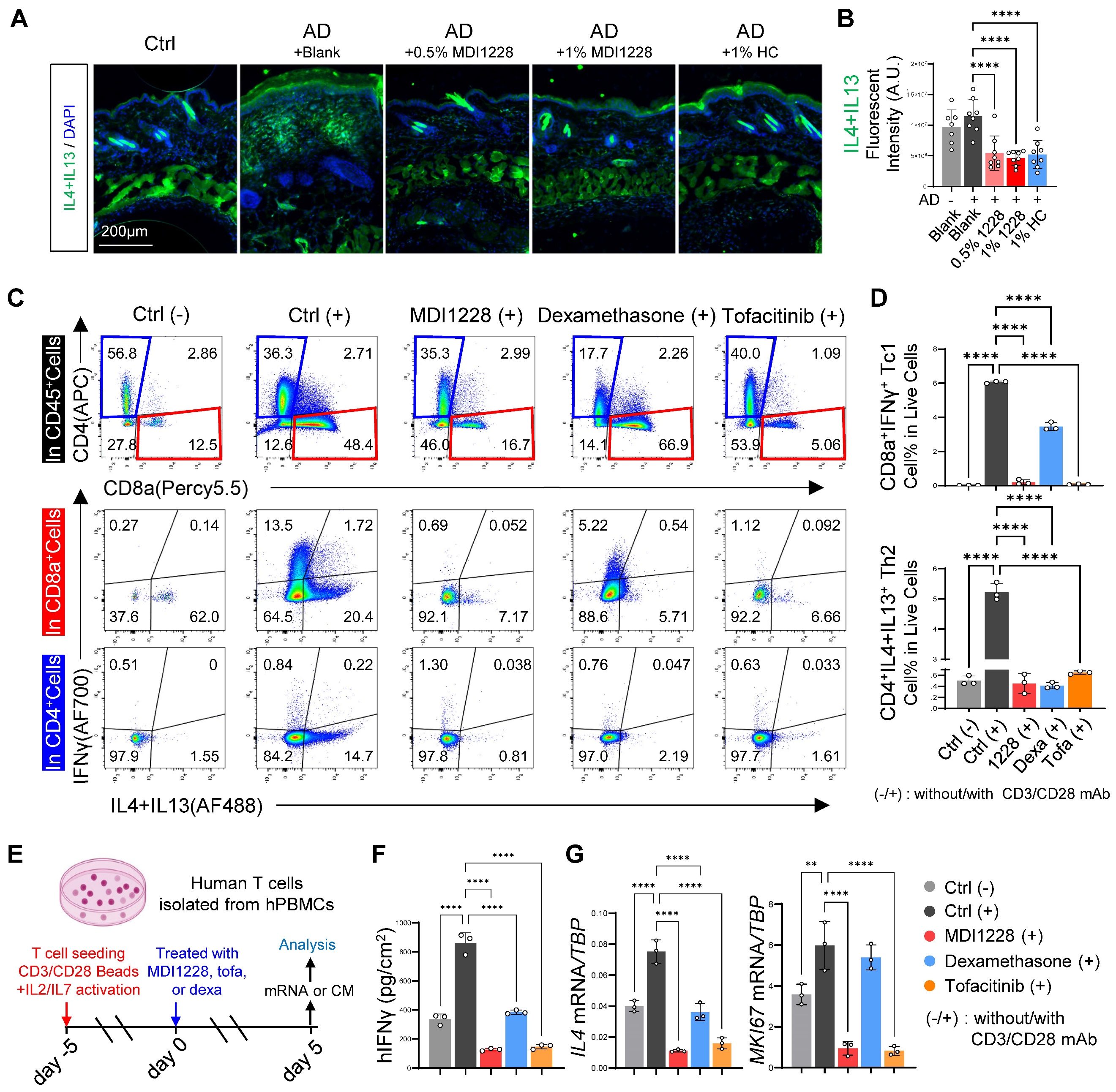
**

**Figure S3. MDI1228 suppresses T cell responses in vitro and in vivo**

(**A-B**) Immunostaining (**A**) and quantified results (**B**) of AD skin sections with anti-IL4+IL13 antibodies (green) and DAPI (blue, n=6/group).

(**C-D**) T cell samples were collected as shown in figure 3G. FACS plots (**C**) and quantified bar graphs (**D**) showing the percentage of CD4+ or CD8+ T cells (up panel), or the percentage of IFNγ+IL4/13- or IFNγ-IL4/13+ in CD4+ (middle panel) and CD8a+ (right panel) T cells in control (without CD3/CD28 mAb), control (with CD3/CD28 mAb), MDI1228, dexamethasone or tofacitinib-treated samples (n=3/group).

(**E**) Overview of the experimental setting. Human T cells were stimulated by IL2 and IL7 with or without CD3/CD28 beads for 5 days, and then were treated with MDI1228, dexamethasone or tofacitinib (1 μM). After 5 days, mRNAs and supernatants were collected for analysis.

(**F-G**) ELISA (**F**) or qRT-PCR (**G**) analysis of the indicated protein/gene expressions in human T cell mRNA/supernatants (n=3/group).

All error bars indicate mean ± SEM. *p < 0.05, **p < 0.01, ***p < 0.001, ****p < 0.0001.

**
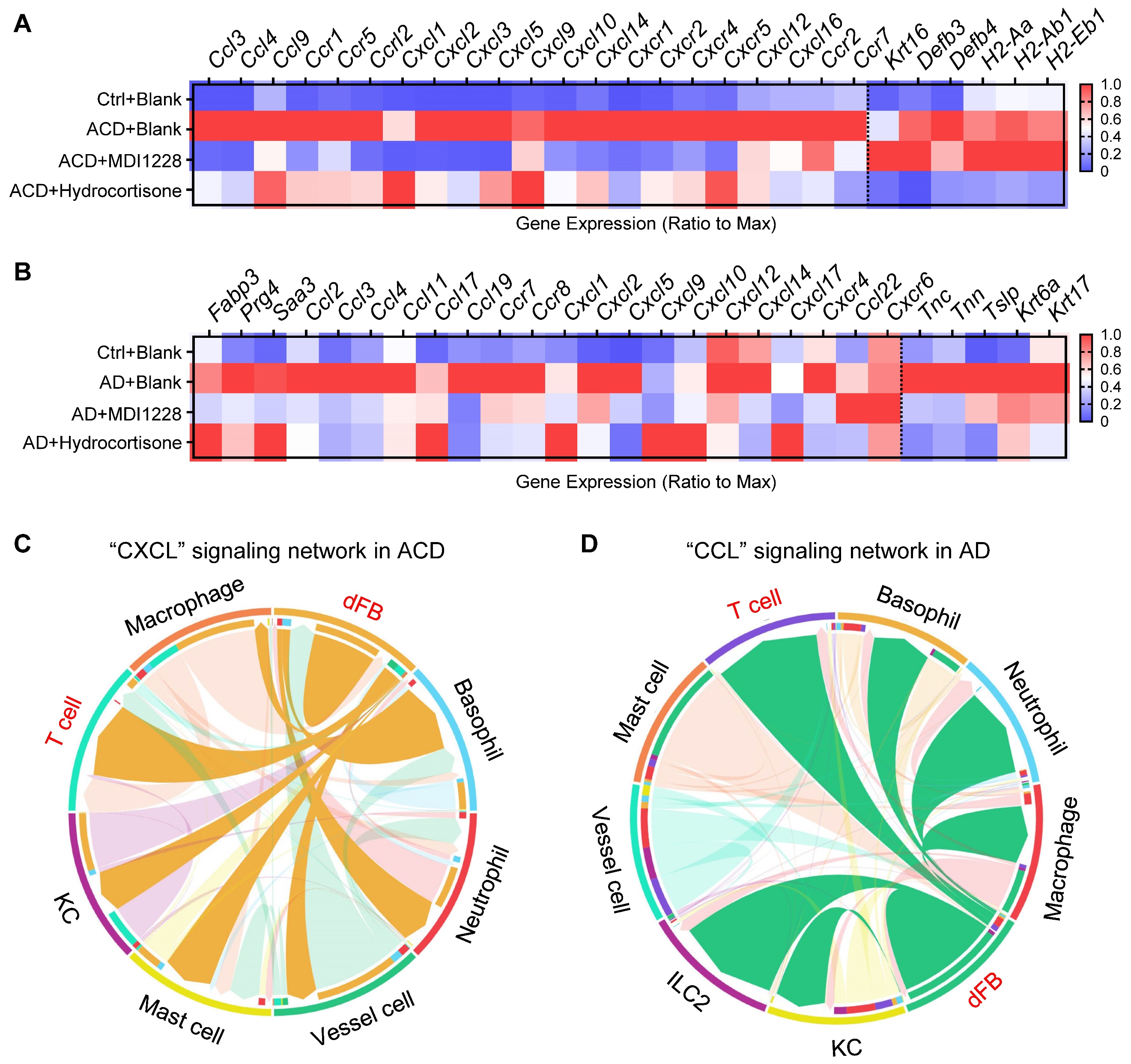
**

**Figure S4. Single‑cell transcriptomic analysis of dFB‑T cell interactions via chemokine signaling axes in ACD and AD**

(**A-B**) Heat maps of selected differentially expressed genes from bulk RNA-seq, showing comparisons of ACD (**A**) or AD (**B**) groups versus other groups.

(**C-D**) Chord diagram showing cell‑pair communication signals mediated solely by a CXCL gene set in ACD skin (**C**) and CCL gene set in AD skin (**D**). Chord width is proportional to the relative interaction strength (p value < 0.05).

**
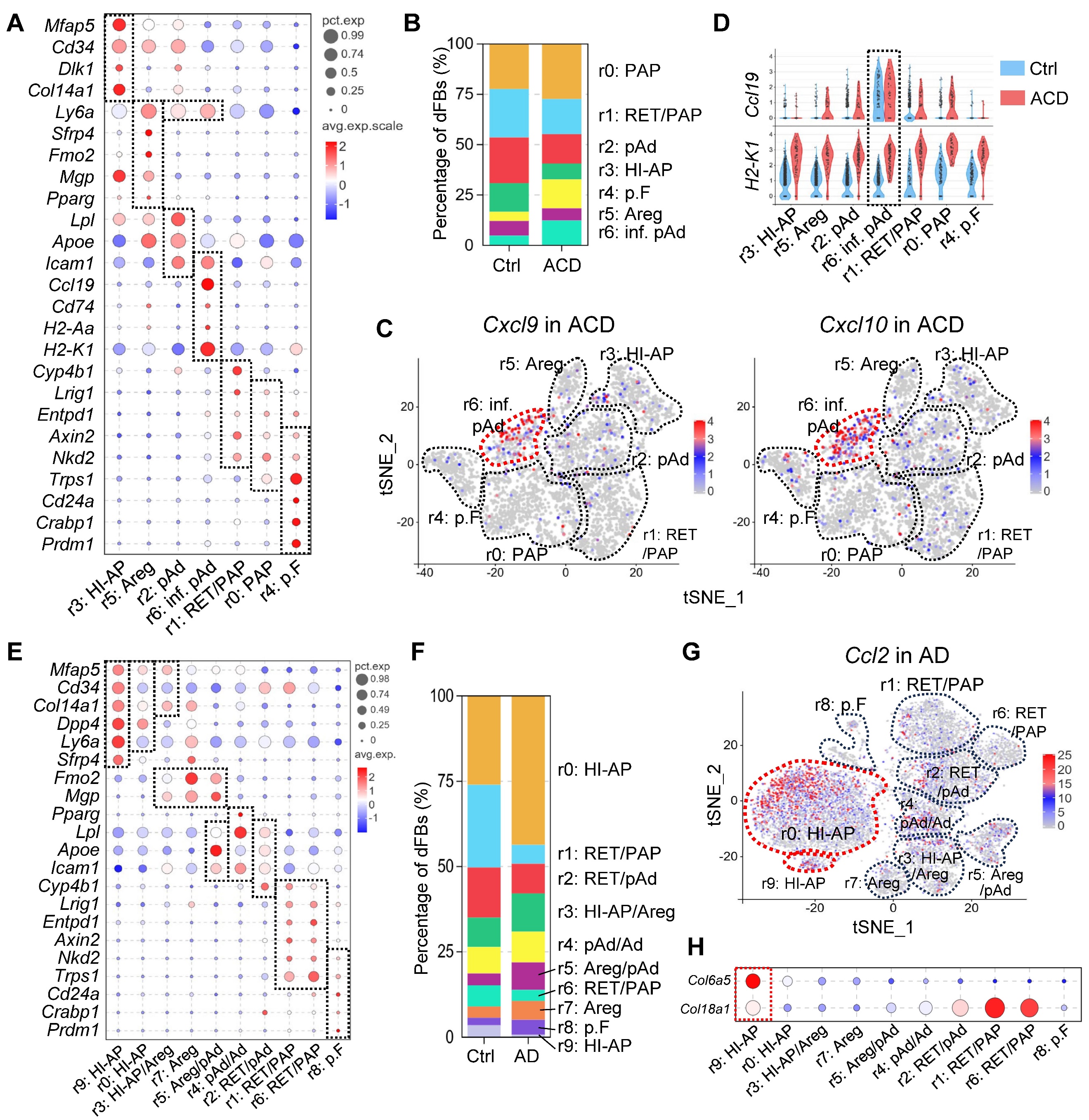
**

**Figure S5. *Pdgfra⁺* dermal fibroblast reclustering reveals the primary sources of pathogenic chemokines in ACD and AD.**

(**A**) tSNE plots showing cell distribution of the *Pdgfra^+^*dermal fibroblasts after re-clustering in ACD.

(**B**) Violin plots showing the expression of indicated genes across various dFB sub-populations in control and ACD samples.

(**C**) Venn diagram comparing the top 100 differential expressed genes upregulated in *CCL19*^+^*CD74*^+^*HLA-DRA*^+^fibroblastic reticular cell-like fibroblasts (Steele et al., 2025), the top 100 genes enriched in r6: inflammatory pre-adipocytes in ACD, and the top 200 IFNγ-inducible genes in primary dFBs (in vitro).

(**D**) tSNE plots showing cell distribution of the *Pdgfra^+^*dermal fibroblasts after re-clustering in AD.

(**E**) Violin plots showing the expression of indicated genes across various dFB sub-populations in control and AD samples.

(**F**) Venn diagram comparing the differential expressed genes upregulated in *CCL19*^+^*CD74*^+^*HLA-DRA*^+^fibroblastic reticular cell-like fibroblasts (Steele et al., 2025), the top 100 genes enriched in merged HI-AP cluster of r0 and r9 in AD, and the top 200 IL4-inducible genes in primary dFBs (in vitro).

Abbreviations: *HI-AP*, hypodermal interstitium adipocyte progenitor; *Areg*, adipogenesis-regulatory cell; *pAd*, pre-adipocyte; *inf. pAd*, inflammatory pre-adipocyte; *RET/PAP*, reticular and/or papillary dFB; *p.F*, peri-follicular dFB.

**
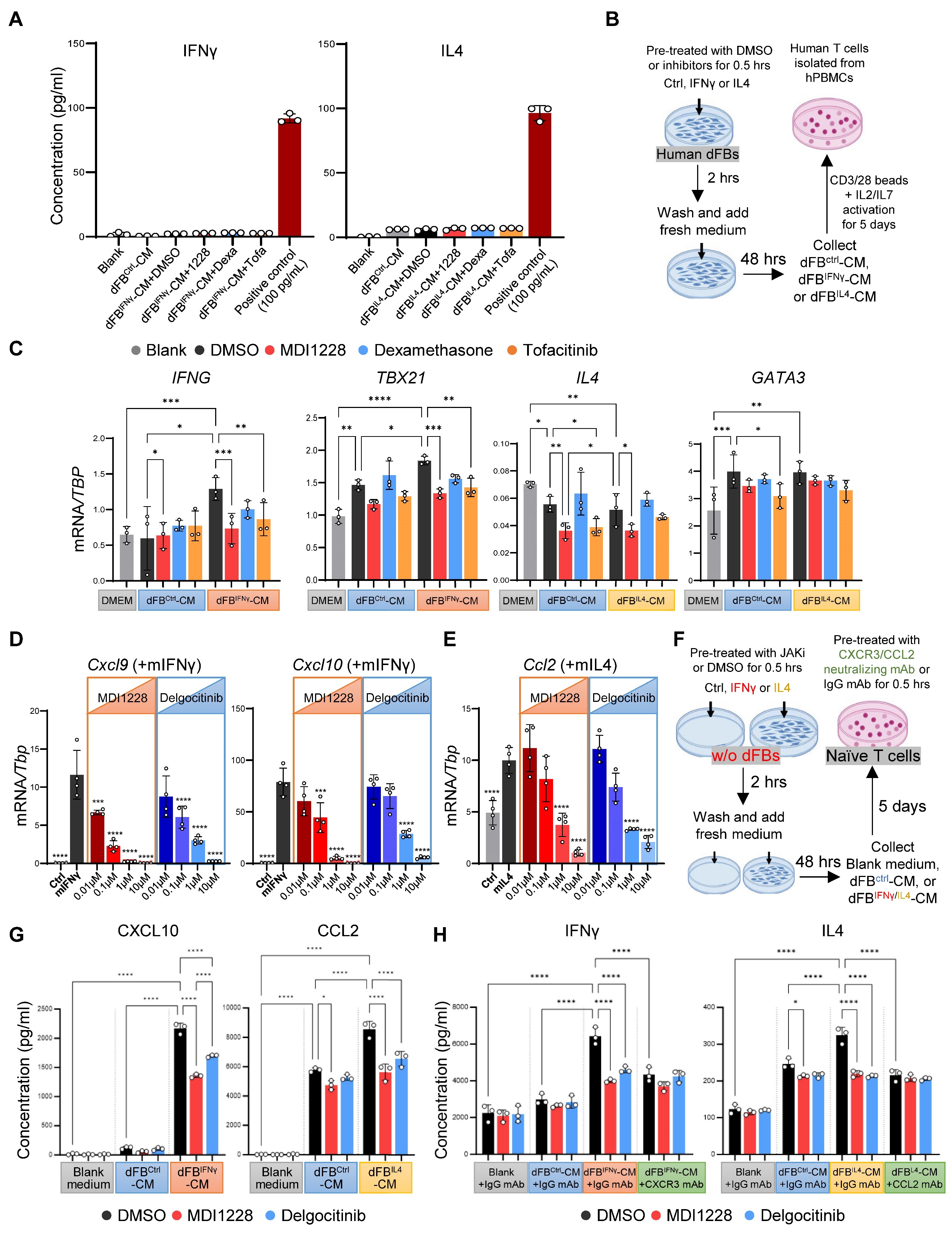
**

**Figure S6. MDI1228 suppresses dFB-derived chemokines to block T cell polarization in vitro**

(**A**) ELISA analysis of IFNγ or IL4 expression levels in dFBCtrl, IFNγ or IL4-CM collected as figure 5D (n=3/group, 100 pg/mL IFNγ or IL4 as positive control).

(**B**) Experiment scheme for collection of IFNγ or IL4-primed hdFB conditioned medium (dFBIFNγ or IL4-CM) or control dFBctrl-CM to stimulate human T cells. HdFBs were pre-treated with 1 μM DMSO, MDI1228, dexamethasone and tofacitinib for 30 mins and then treated with hIFNγ, hIL4 or PBS control for 2 hrs. Cells were washed twice with PBS then replenished with fresh medium without IFNγ or IL4 for additional 48 hours, and CM was collected for the 5-day-co-culture with T cells (n=3/group).

(**C**) qRT-PCR analysis of the indicated protein/gene expressions in human T cell mRNA (n=3/group).

(**D-E**) Mouse dFBs were pre-treated with MDI1228 or delgocitinib (0.01μM, 0.1μM, 1μM, or 10μM) for 30 mins and then treated with mIFNγ (**D**) or mIL4 (**E**) for 24 hrs. Control or cytokine-treated samples were subjected to qRT-PCR analysis of indicated gene mRNA expression (n=4/group). Comparisons were performed between cytokine-stimulated group and each other group via one-way analysis of variance.

(**F**) Experiment scheme for collection of IFNγ or IL4-primed dFB conditioned medium (dFB^IFNγ or IL4^-CM) or control dFB^ctrl^-CM to stimulate naïve T cells. Primary dFBs were pre-treated with 5μM DMSO, MDI1228, or delgocitinib for 30 mins and then treated with IFNγ or PBS control for 2 hrs. Cells were washed twice with PBS then replenished with fresh medium without IFNγ or IL4 for additional 48 hrs. As a drug-only washing control, cell-free wells were supplemented with DMSO, MDI1228, or delgocitinib and subjected to the same washing and medium change steps. Finally, cell-free blank control or dFB-CMs were collected for the 5-day-co-culture with T cells (n=3/group).

(**G**) ELISA analysis of CXCL10 or CCL11 expression levels in blank controls or dFB^Ctrl, IFNγ or IL4^-CMs (n=3/group).

(**H**) Naïve T lymphocytes stimulated with CD3/28-Ab were treated with blank control, dFB^ctrl^-CM, dFB^IFNγ^-CM or dFB^IL4^-CM, and cell supernatants were collected for ELISA analysis of IFNγ or IL4 protein expression (n=3/group).

All error bars indicate mean ± SEM. *p < 0.05, **p < 0.01, ***p < 0.001, ****p < 0.0001.

**
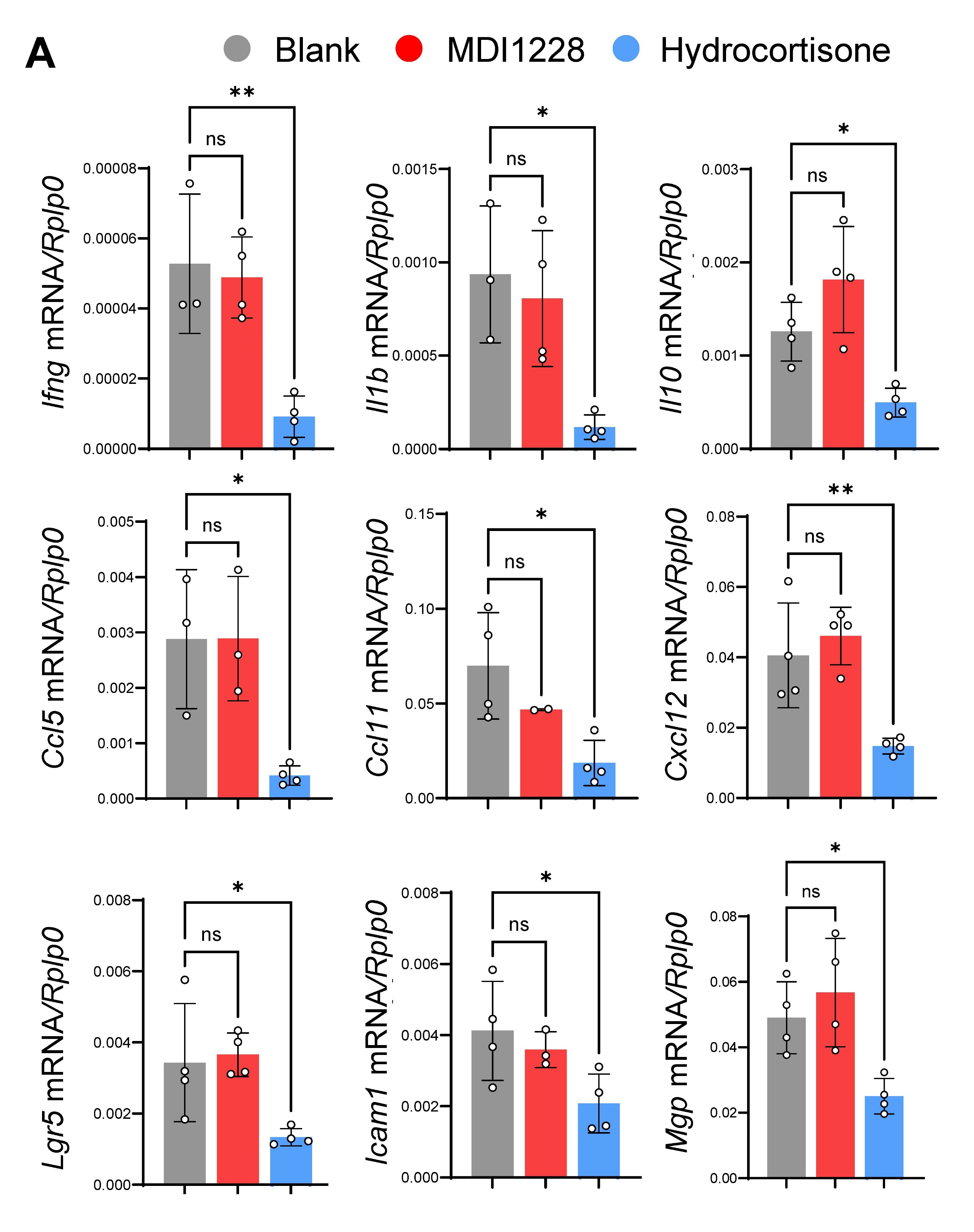
**

**Figure S7. Repeated topical MDI1228 administration for 14 days demonstrates superior safety compared to glucocorticoids**

(**A**) qRT-PCR analysis of the indicated mRNA expression levels of mouse skin for each group (n=3~4/group).

All error bars indicate mean ± SEM. *p < 0.05, **p < 0.01, ***p < 0.001, ****p < 0.0001.

# Supplementary Tables

**Supplementary table 1. List of primers used for qRT-PCR of mouse genes.**

| **Gene** | **Strand** | **Primer sequence** |
| --- | --- | --- |
| ***Tbp*** | Forward | CCTTGTACCCTTCACCAATGAC |
|  | Reverse | ACAGCCAAGATTCACGGTAGA |
| ***Rplp0*** | Forward | TGAGATTCGGGATATGCTGTTGG |
|  | Reverse | CGGGTCCTAGACCAGTGTTCT |
| ***Cd4*** | Forward | CTAGCTGTCACTCAAGGGAAGA |
|  | Reverse | CGAAGGCGAACCTCCTCTAA |
| ***Cd8a*** | Forward | GACCGGATTGGACTTCGCCT |
|  | Reverse | GACTAGCGGCCTGGGACATT |
| ***Ifng*** | Forward | GCCACGGCACAGTCATTGA |
|  | Reverse | TGCTGATGGCCTGATTGTCTT |
| ***Il4*** | Forward | GAGCCATATCCACGGATGCGAC |
|  | Reverse | ATGCGAAGCACCTTGGAAGCCC |
| ***Il13*** | Forward | TGCTTGCCTTGGTGGTCTCGC |
|  | Reverse | GCGGCCAGGTCCACACTCCA |
| ***Il17a*** | Forward | ACGCGCAAACATGAGTCCAGGG |
|  | Reverse | TGAGGGATGATCGCTGCTGCCT |
| ***Mcpt8*** | Forward | AACGCTGAAGGAGGGGAAATCA |
|  | Reverse | TTGCCACCAGGAAACCACCA |
| ***Gzma*** | Forward | TTGGAGGAGACACGGTTGTT |
|  | Reverse | TTGATTGAGTGAGCCCCAAGA |
| ***Cxcl10*** | Forward | CCACGTGTTGAGATCATTGCCACG |
|  | Reverse | ATCCATCGCAGCACCGGGGT |
| ***Cxcr3*** | Forward | TACCTTGAGGTTAGTGAACGTCA |
|  | Reverse | CGCTCTCGTTTTCCCCATAATC |
| ***Ccl2*** | Forward | CACAGTTGCCGGCTGGAGCA |
|  | Reverse | CAGCAGGTGAGTGGGGCGTT |
| ***Ccr2*** | Forward | ATCCACGGCATACTATCAACATC |
|  | Reverse | CAAGGCTCACCATCATCGTAG |
| ***Il1b*** | Forward | GAAATGCCACCTTTTGACAGTG |
|  | Reverse | TGGATGCTCTCATCAGGACAG |
| ***Ccl5*** | Forward | GCTGCTTTGCCTACCTCTCC |
|  | Reverse | TCGAGTGACAAACACGACTGC |
| ***Il10*** | Forward | GGCGCTGTCATCGATTTCTCCCC |
|  | Reverse | GGCCTTGTAGACACCTTGGTCTTGG |
| ***Pparg2*** | Forward | TCGCTGATGCACTGCCTATG |
|  | Reverse | GAGAGGTCCACAGAGCTGATT |
| ***Icam1*** | Forward | TCCGCTACCATCACCGTGTAT |
|  | Reverse | TAGCCAGCACCGTGAATGTG |
| ***Cxcl12*** | Forward | TGCATCAGTGACGGTAAACCA |
|  | Reverse | TTCTTCAGCCGTGCAACAATC |
| ***Mgp*** | Forward | GGCAACCCTGTGCTACGAAT |
|  | Reverse | CCTGGACTCTCTTTTGGGCTTTA |
| ***Lgr5*** | Forward | CCTACTCGAAGACTTACCCAGT |
|  | Reverse | GCATTGGGGTGAATGATAGCA |
| ***hTBP*** | Forward | CCCGAAACGCCGAATATAATCC |
|  | Reverse | AATCAGTGCCGTGGTTCGTG |
| ***hCXCL9*** | Forward | TGAGAAAGGGTCGCTGTTCC |
|  | Reverse | TCACATCTGCTGAATCTGGGT |
| ***hCXCL10*** | Forward | TGCCATTCTGATTTGCTGCC |
|  | Reverse | GCCTTCGATTCTGGATTCAG |
| ***hCCL2*** | Forward | GAAAGTCTCTGCCGCCCTT |
|  | Reverse | CTTCTTTGGGACACTTGCTGC |
| ***hTBX21*** | Forward | CCACCTGTTGTGGTCCAAGT |
|  | Reverse | CCCGGCCACAGTAAATGACA |
| ***hGATA3*** | Forward | TACGTGCCCGAGTACAGCTC |
|  | Reverse | CGTTGCACAGGTAGTGTCCC |
| ***hMKI67*** | Forward | CCTGTACGGCTAAAACATGGAG |
|  | Reverse | GAATAGGCCTTGGAATCTTGAGC |

**Supplementary table 2. Kinase inhibition profile of 0.2 μM MDI1228.**

| **Kinase name** | **Inhibition (%) @ 0.2 μM** |
| --- | --- |
| ABL_1mM | 49.5 |
| ALK_1mM | 83.3 |
| ALK [C1156Y]_1mM | 80.8 |
| ALK [G1202R]_1mM | 27.9 |
| ALK [G1269A]_1mM | 53.3 |
| ALK [L1152insT]_1mM | 69.6 |
| ALK [R1275Q]_1mM | 77.6 |
| AXL_1mM | 5.6 |
| BLK_1mM | 46.6 |
| BMX_1mM | 66.1 |
| BRK_1mM | 64.4 |
| BTK_1mM | -8.9 |
| BTK [C481S]_1mM | -5.5 |
| CSK_1mM | 1.3 |
| EGFR_1mM | 7.0 |
| EGFR [d746-750]_1mM | -1.9 |
| EGFR [d746-750/T790M]_1mM | -6.4 |
| EGFR [L858R]_1mM | -3.6 |
| EGFR [L861Q]_1mM | -3.4 |
| EGFR [T790M]_1mM | -4.0 |
| EGFR [T790M/L858R]_1mM | -2.0 |
| FAK_1mM | -5.0 |
| FER_1mM | 5.8 |
| FES_1mM | 2.5 |
| FGFR1_1mM | 2.8 |
| FGFR2_1mM | 90.7 |
| FGFR2 [V564I]_1mM | 11.8 |
| FGFR3_1mM | 14.7 |
| FGFR4_1mM | 15.8 |
| FGFR4 [V550E]_1mM | -0.6 |
| FGFR4 [V550L]_1mM | -0.7 |
| FGR_1mM | 56.0 |
| FRK_1mM | 7.6 |
| FYN [isoform a]_1mM | 49.4 |
| FYN [isoform b]_1mM | 26.2 |
| HCK_1mM | 20.1 |
| HER2_1mM | 8.5 |
| HER4_1mM | 21.2 |
| IGF1R_1mM | -1.3 |
| INSR_1mM | -1.8 |
| IRR_1mM | -0.7 |
| ITK_1mM | 19.8 |
| JAK1_1mM | 100.6 |
| JAK2_1mM | 99.8 |
| JAK3_1mM | 98.6 |
| KIT_1mM | 18.0 |
| KIT [D816E]_1mM | 8.6 |
| KIT [D816V]_1mM | 11.9 |
| KIT [D816Y]_1mM | 11.6 |
| KIT [T670I]_1mM | 9.8 |
| KIT [V560G]_1mM | 20.5 |
| KIT [V654A]_1mM | 0.2 |
| LTK_1mM | 90.2 |
| MER_1mM | 18.8 |
| MET_1mM | 5.5 |
| MET [D1228H]_1mM | 1.5 |
| MET [M1250T]_1mM | 0.0 |
| MET [Y1235D]_1mM | 12.7 |
| MUSK_1mM | 56.6 |
| PDGFRα_1mM | 42.6 |
| PDGFRα [D842V]_1mM | 28.9 |
| PDGFRα [T674I]_1mM | 20.6 |
| PDGFRα [V561D]_1mM | 43.4 |
| PDGFRβ_1mM | 47.1 |
| PYK2_1mM | 25.9 |
| RET_1mM | 64.9 |
| RON_1mM | 20.9 |
| ROS_1mM | 10.0 |
| SRM_1mM | 15.4 |
| SYK_1mM | 70.3 |
| TEC_1mM | 12.6 |
| TNK1_1mM | 20.2 |
| TXK_1mM | 19.8 |
| TYK2_1mM | 96.8 |
| YES_1mM | 29.2 |
| ZAP70_1mM | 1.6 |
| AKT1_1mM | -12.6 |
| AMPKα1/β1/γ1_1mM | 6.5 |
| AurA_1mM | 1.5 |
| AurB_1mM | 0.1 |
| AurC_1mM | 3.3 |
| BRSK1_1mM | 19.3 |
| CaMK1α | 2.9 |
| CaMK4_1mM | 0.1 |
| CDC2/CycB1_1mM | 27.6 |
| CDC7/ASK_1mM | -8.7 |
| CDK2/CycA2_1mM | 53.5 |
| CDK2/CycE1_1mM | 39.6 |
| CDK3/CycE1 | 70.7 |
| CDK4/CycD3_1mM | 83.5 |
| CDK5/p25_1mM | 15.7 |
| CDK6/CycD3_1mM | 70.0 |
| CDK7/CycH/MAT1_1mM | 0.3 |
| CDK9/CycT1_1mM | 25.4 |
| CHK1_1mM | 12.5 |
| CHK2_1mM | -4.2 |
| CK1α_1mM | 0.4 |
| CK1ε_1mM | -1.5 |
| CK2α1/β_1mM | -0.6 |
| CLK1_1mM | 49.7 |
| CLK2_1mM | 67.3 |
| DAPK1_1mM | -3.2 |
| DYRK1A_1mM | 7.1 |
| DYRK1B_1mM | 33.0 |
| Erk1_1mM | -18.6 |
| Erk2_1mM | -6.5 |
| GSK3α_1mM | 9.9 |
| GSK3β_1mM | 15.3 |
| HGK_1mM | 15.0 |
| HIPK4_1mM | 2.4 |
| IKKβ_1mM | -1.3 |
| IRAK4 | 29.3 |
| JNK1_1mM | -3.0 |
| JNK2_1mM | 0.4 |
| JNK3_1mM | -2.8 |
| MAPKAPK2_1mM | -14.6 |
| MINK_1mM | 23.4 |
| MST1_1mM | 12.9 |
| NEK1_1mM | 23.2 |
| NEK2_1mM | 4.8 |
| NEK6_1mM | -2.8 |
| NEK7_1mM | 2.5 |
| NEK9_1mM | 25.7 |
| p38α_1mM | -2.4 |
| p38β_1mM | -1.6 |
| p38γ_1mM | 0.3 |
| p38δ_1mM | -3.4 |
| p70S6K_1mM | 21.6 |
| PAK2_1mM | -2.5 |
| PBK_1mM | -2.2 |
| PDK1_1mM | -2.1 |
| PIM1_1mM | 3.3 |
| PIM2_1mM | -4.9 |
| PKACα_1mM | -24.6 |
| PKCα_1mM | 39.1 |
| PKCε_1mM | 13.5 |
| PKD2_1mM | -21.0 |
| PLK1_1mM | -0.8 |
| PLK3_1mM | 3.1 |
| QIK_1mM | 22.1 |
| ROCK1_1mM | -1.0 |
| RSK1_1mM | 0.5 |
| RSK3_1mM | -3.4 |
| RSK4_1mM | 5.7 |
| SGK_1mM | -4.0 |
| SIK_1mM | 39.4 |
| TNIK_1mM | 30.3 |
| TSSK1_1mM | -2.2 |

**Supplementary table 3. Body weight, hematology and liver function parameters in rats after 14-day repeated topical application of MDI1228.**

| **Dose (mg/kg/day)** | **Sex** | **Body weight Day 1 (g)** | **Body weight Day 14 (g)** | **Body weight gain (g)** | **WBC (×10^9/L)** | **ALT (U/L)** | **AST (U/L)** |
| --- | --- | --- | --- | --- | --- | --- | --- |
| 0 (Control) | Male | 290.8 ± 10.9 | 338.4 ± 10.0 | 47.6 ± 11.5 | 12.37 ± 0.43 | 37.0 ± 4.7 | 97.4 ± 4.8 |
|  | Female | 215.0 ± 9.5 | 243.6 ± 17.0 | 28.6 ± 10.0 | 11.13 ± 5.25 | 29.4 ± 4.0 | 97.2 ± 14.2 |
| 20 | Male | 295.7 ± 14.7 | 344.5 ± 35.2 | 48.8 ± 23.4 | 14.62 ± 2.04 | 40.6 ± 7.4 | 105.4 ± 10.2 |
|  | Female | 213.9 ± 8.5 | 236.7 ± 16.3 | 22.8 ± 10.8 | 15.32 ± 4.41 | 30.8 ± 3.8 | 107.4 ± 20.3 |
| 40 | Male | 291.8 ± 7.5 | 339.0 ± 18.2 | 47.2 ± 11.3 | 13.70 ± 4.84 | 35.6 ± 5.3 | 98.8 ± 3.8 |
|  | Female | 215.4 ± 10.5 | 234.6 ± 11.5 | 19.2 ± 2.4 | 11.62 ± 3.52 | 38.2 ± 2.9 | 101.2 ± 9.7 |
| 120 | Male | 294.5 ± 11.5 | 350.2 ± 20.8 | 55.6 ± 12.4 | 13.60 ± 3.12 | 41.0 ± 6.3 | 108.4 ± 13.1 |
|  | Female | 216.3 ± 12.5 | 238.4 ± 7.1 | 22.0 ± 14.9 | 11.61 ± 3.04 | 40.4 ± 10.1 | 115.4 ± 19.3 |

Data are presented as mean ± SD. BW, body weight; Body weight gain, calculated as (Body weight on Day 14) - (Body weight on Day 1); WBC, white blood cell count; ALT, alanine aminotransferase; AST, aspartate aminotransferase.

**Supplementary table 4. Absolute and relative organ weights in rats after 14-day repeated topical application of MDI1228 (Day 15).**

| **Dose (mg/kg/day)** | **Sex** | **Heart (g)** | **Heart/**  **BW (%)** | **Liver (g)** | **Liver/**  **BW (%)** | **Spleen (g)** | **Spleen/BW (%)** | **Kidney (g)** | **Kidney/BW (%)** |
| --- | --- | --- | --- | --- | --- | --- | --- | --- | --- |
| 0 (Control) | Male | 1.374  ± 0.156 | 0.438  ± 0.042 | 9.699  ± 0.487 | 3.092  ± 0.084 | 0.722  ± 0.109 | 0.230  ± 0.034 | 2.549  ± 0.145 | 0.813  ± 0.034 |
|  | Female | 0.980  ± 0.040 | 0.445  ± 0.022 | 7.507  ± 0.694 | 3.402  ± 0.178 | 0.548  ± 0.090 | 0.248  ± 0.030 | 1.924  ± 0.126 | 0.875  ± 0.081 |
| 20 | Male | 1.356  ± 0.127 | 0.426  ± 0.019 | 9.798  ± 1.734 | 3.058  ± 0.231 | 0.685  ± 0.155 | 0.215  ± 0.039 | 2.574  ± 0.460 | 0.803  ± 0.063 |
|  | Female | 0.965  ± 0.058 | 0.450  ± 0.038 | 7.157  ± 0.471 | 3.332  ± 0.164 | 0.496  ± 0.073 | 0.231  ± 0.033 | 1.771  ± 0.236 | 0.824  ± 0.092 |
| 40 | Male | 1.312  ± 0.108 | 0.419  ± 0.025 | 9.793  ± 0.692 | 3.127  ± 0.188 | 0.684  ± 0.138 | 0.218  ± 0.041 | 2.526  ± 0.154 | 0.806  ± 0.013 |
|  | Female | 0.994  ± 0.140 | 0.466  ± 0.052 | 7.061  ± 0.666 | 3.313  ± 0.179 | 0.485  ± 0.038 | 0.228  ± 0.018 | 1.756  ± 0.087 | 0.826  ± 0.042 |
| 120 | Male | 1.341  ± 0.169 | 0.412  ± 0.031 | 10.466  ± 0.738 | 3.228  ± 0.184 | 0.718  ± 0.122 | 0.221  ± 0.032 | 2.698  ± 0.233 | 0.831  ± 0.048 |
|  | Female | 0.941  ± 0.103 | 0.437  ± 0.060 | 7.009  ± 0.342 | 3.251  ± 0.249 | 0.438  ± 0.019* | 0.203  ± 0.007* | 1.760  ± 0.107 | 0.817  ± 0.077 |

Data are presented as mean ± SD. BW, body weight (g) measured on Day 15 before necropsy; Heart/BW (%) = (Heart weight (g) / Body weight (g)) × 100, similarly for Liver/BW (%), Spleen/BW (%), Kidney/BW (%).

**Supplementary table 5. Local skin reactions and histopathological parameters in rats after 14-day repeated topical application of MDI1228.**

| **Dose (mg/kg/day)** | **Erythema/edema (Draize score 0-4)** | **Gross necropsy findings** | **Histopathology (H&E of application site)** |
| --- | --- | --- | --- |
| 0 | None (score 0) | No abnormalities | Not performed (not required for control) |
| 20 | None (score 0) | One male had focal yellow discoloration of epididymis (incidental, not drug-related) | Not performed (no gross lesions) |
| 40 | None (score 0) | No abnormalities | Not performed |
| 120 | None (score 0) | One female had bladder calculus and mucosal thickening (incidental, not drug-related) | Not performed |

Erythema/edema, assessed by Draize scoring system (0 = no reaction, 4 = severe); Gross necropsy findings, macroscopic examination of all major organs; Histopathology (H&E of application site), hematoxylin and eosin staining of skin at the application site.

**Supplementary table 6. List of Abbreviations.**

| **Abbreviations** | **Definitions** |
| --- | --- |
| ACD | Allergic Contact Dermatitis |
| AD | Atopic Dermatitis |
| ALT | Alanine Aminotransferase |
| AP-1 | Activator Protein 1 |
| Areg | Adipogenesis-Regulatory Cell |
| AST | Aspartate Aminotransferase |
| AUC_last_ | Area Under the Concentration-Time Curve from the Start to the Last Dosing Time Point |
| BSA | Body Surface Area |
| CCL | C-C Motif Chemokine Ligand |
| CM | Conditioned Medium |
| C_max_ | Maximum Plasma Concentration |
| CXCL | C-X-C Motif Chemokine Ligand |
| dFB | Dermal Fibroblast |
| DMEM | Dulbecco's Modified Eagle Medium |
| DMSO | Dimethyl Sulfoxide |
| DNFB | 2,4-Dinitrofluorobenzene |
| dWAT | Dermal White Adipose Tissue |
| EASI | Eczema Area and Severity Index |
| ELISA | Enzyme-Linked Immunosorbent Assay |
| eWAT | Epididymal White Adipose Tissue |
| FBS | Fetal Bovine Serum |
| FDA | U.S. Food and Drug Administration |
| FRC | Fibroblastic Reticular Cell |
| GC | Glucocorticoid |
| GSEA | Gene Set Enrichment Analysis |
| H&E | Hematoxylin and Eosin |
| HC | Hydrocortisone |
| HI-AP | Hypodermal Interstitium Adipocyte Progenitor |
| IC_50_ | Half-Maximal Inhibitory Concentration |
| IFNγ | Interferon-Gamma |
| IHC | Immunohistochemistry |
| IL | Interleukin |
| inf. pAd | Inflammatory Pre-Adipocyte |
| JAK | Janus Kinase |
| KEGG | Kyoto Encyclopedia of Genes and Genomes |
| LC-MS/MS | Liquid Chromatography-Tandem Mass Spectrometry |
| MC903 | Calcipotriol |
| MTD | Maximum Tolerated Dose |
| NK | Natural Killer |
| p.F | Peri-Follicular Dermal Fibroblast |
| pAd | Pre-Adipocyte |
| qRT-PCR | Quantitative Reverse Transcription-Polymerase Chain Reaction |
| RET/PAP | Reticular and/or Papillary Dermal Fibroblast |
| RNA-seq | RNA Sequencing |
| RTK | Receptor Tyrosine Kinase |
| SCORAD | SCORing Atopic Dermatitis |
| scRNA-seq | Single-Cell RNA Sequencing |
| SD | Standard Deviation |
| SEM | Standard Error of the Mean |
| SPF | Specific Pathogen-Free |
| STAT | Signal Transducer and Activator of Transcription |
| Tc | Cytotoxic T |
| Th | T Helper |
| tSNE | T-Distributed Stochastic Neighbor Embedding |
| TYK2 | Tyrosine Kinase 2 |
| WBC | White Blood Cell |
